# Thromboxane Mobilizes Insect Blood Cells to Infection Foci

**DOI:** 10.3389/fimmu.2021.791319

**Published:** 2021-12-20

**Authors:** Miltan Chandra Roy, Kiwoong Nam, Jaesu Kim, David Stanley, Yonggyun Kim

**Affiliations:** ^1^ Department of Plant Medicals, Andong National University, Andong, South Korea; ^2^ DGIMI, Univ Montpellier, INRAE, Montpellier, France; ^3^ Department of Agricultural Convergence Technology, Jeonbuk National University, Jeonju, South Korea; ^4^ Biological Control of Insect Research Laboratory, United States Department of Agriculture-Agricultural Research Station (USDA/ARS), Columbia, MO, United States

**Keywords:** insect, fungi, thromboxane, *Spodoptera exigua*, hemocyte

## Abstract

Innate immune responses are effective for insect survival to defend against entomopathogens including a fungal pathogen, *Metarhizium rileyi*, that infects a lepidopteran *Spodoptera exigua*. In particular, the fungal virulence was attenuated by cellular immune responses, in which the conidia were phagocytosed by hemocytes (insect blood cells) and hyphal growth was inhibited by hemocyte encapsulation. However, the chemokine signal to drive hemocytes to the infection foci was little understood. The hemocyte behaviors appeared to be guided by a Ca^2+^ signal stimulating cell aggregation to the infection foci. The induction of the Ca^2+^ signal was significantly inhibited by the cyclooxygenase (COX) inhibitor. Under the inhibitory condition, the addition of thromboxane A_2_ or B_2_ (TXA_2_ or TXB_2_) among COX products was the most effective to recover the Ca^2+^ signal and hemocyte aggregation. TXB_2_ alone induced a microaggregation behavior of hemocytes under *in vitro* conditions. Indeed, TXB_2_ titer was significantly increased in the plasma of the infected larvae. The elevated TXB_2_ level was further supported by the induction of phospholipase A_2_ (PLA_2_) activity in the hemocytes and subsequent up-regulation of COX-like peroxinectins (*SePOX-F* and *SePOX-H*) in response to the fungal infection. Finally, the expression of a thromboxane synthase (*Se-TXAS*) gene was highly expressed in the hemocytes. RNA interference (RNAi) of *Se-TXAS* expression inhibited the Ca^2+^ signal and hemocyte aggregation around fungal hyphae, which were rescued by the addition of TXB_2_. Without any ortholog to mammalian thromboxane receptors, a prostaglandin receptor was essential to mediate TXB_2_ signal to elevate the Ca^2+^ signal and mediate hemocyte aggregation behavior. Specific inhibitor assays suggest that the downstream signal after binding TXB_2_ to the receptor follows the Ca^2+^-induced Ca^2+^ release pathway from the endoplasmic reticulum of the hemocytes. These results suggest that hemocyte aggregation induced by the fungal infection is triggered by TXB_2_ *via* a Ca^2+^ signal through a PG receptor.

## Introduction

Insect innate immunity includes cellular and humoral immune responses to prokaryotic and eukaryotic pathogens and parasites (1). Cellular responses are acutely induced and performed by mesodermal blood cells called hemocytes. Cellular actions include phagocytosis, encapsulation, and nodulation, depending on the type of invading pathogens (2, 3). In humoral immunity, antimicrobial peptides (AMPs) are produced and secreted by fat body and some hemocytes into hemolymph circulation to remove the residual pathogens after the cellular immune defense (4). Immune mediators such as cytokines and eicosanoids induce cellular and humoral immune responses to effectively defend against entomopathogens in insects (5, 6).

Eicosanoids are a group of oxygenated C20 polyunsaturated fatty acids including prostaglandins (PGs) that mediate cellular and humoral responses against various pathogens in insects as well as mammals (7). PGs are usually derived from arachidonic acid (AA: 5,8,11,14-eicosatetraenoic acid) by cyclooxygenase (COX) (8). However, terrestrial insects lack AA in phospholipids and thus transform C18 linoleic acid to AA by elongase and desaturase (9, 10). AA is then oxygenated by the dioxygenase activity of cyclooxygenase (COX) to form PGH_2_, which is isomerized by various cell-specific PG synthases to form prostanoids (11). More specifically, a prostanoid, thromboxane A_2_ (TXA_2_) is formed by TXA_2_ synthase (TXAS), a member of the cytochrome P450 epoxygenase superfamily. TXA_2_ exerts its biological activity through a G protein-coupled receptor called TP (12).

Thromboxanes act in blood clotting in mammals by reducing blood flow through vasoconstriction and driving platelets near to the clotting site (13). A similar blood clotting process has been reported in *Drosophila melanogaster* by demonstrating the presence coagulation factors commonly found in humans (14). Eicosanoids mediate the wound healing process since a *Drosophila* line mutant in PLA_2_ suffers hemolymph coagulation failures (15). Although this study did not identify specific eicosanoids, prostanoids like thromboxanes might be associated with wound healing in *Drosophila*. Two thromboxanes (TXA_2_ and TXB_2_) mediate immune responses in a model lepidopteran insect, *Spodoptera exigua* (16). Chemical identification of TXB_2_ in fat body and the presence of its biosynthetic gene (*Se-TXAS*) supported the physiological role of these thromboxanes.

Various roles of PGs in immune mediation have been unraveled in selected insect species. PGs mobilize sessile hemocytes to increase the number of circulatory hemocytes in plasma in *S. exigua* (17). In *Manduca sexta*, PGs promote the migration of hemocytes to infection foci (18). *Anopheles* mosquito midgut cells produce and release PGE_2_, which attracts hemocytes and establishes a systemic cellular immune response to the malarial pathogen (19). In the infection site, PGs activate hemocytes to extend cytoplasm to induce hemocyte-spreading behavior, which is required for phagocytosis (20), nodulation, and encapsulation (21, 22). PGs also induce the release of prophenoloxidase (PPO) from oenocytoids (a type of hemocytes) into hemolymph, where it is activated to phenoloxidase (PO) for melanin formation around nodules and encapsulated parasitoids (23). PGs mediate the synthesis of various AMPs among insect species (20, 24). Despite the multi-functional roles of PGs, we began to understand their respective physiological functions because thromboxanes do not mediate female reproduction unlike other PGs (PGD_2_ and PGE_2_) that mediate oocyte development in *Drosophila melanogaster* and *S. exigua* (25–27). These suggest that prostanoids may mediate their specific physiological processes within general immune responses, such as nodulation.

Based on the roles of thromboxanes in platelet aggregation in the process of mammalian blood clotting, we tested the physiological role of thromboxanes in recruiting hemocytes to infection foci in insects using *S. exigua*. To explain the cellular behavior with respect to intracellular processes, we assessed Ca^2+^ signaling in response to thromboxanes in *S. exigua*.

## Materials and Methods

### Insect Rearing

Larvae of two lepidopteran species (*S. exigua* and *Plutella xylostella*) were collected from Welsh onion (*Allium fistulosum* L.) and Chinese cabbage (*Vigna angularis*) fields in Andong, Korea, respectively. Coleopteran (*Tenebrio molitor*) larvae were provided by Bio Utility (Andong, Korea). Insects were reared in a laboratory under our standard conditions of 25 ± 2°C temperature, 16:8 h (L:D) photoperiod, and 60 ± 5% relative humidity (RH). *S. exigua* larvae were reared on an artificial diet (28) and underwent five larval instars (L1-L5). Larvae of *P. xylostella* were reared on fresh cabbage leaves and underwent four larval instars (L1-L4). Approximately 3.5 cm body length *T*. *molitor* larvae were used for pathogenicity testing.

### Chemicals

Arachidonic acid (AA: 5,8,11,14-eicosatetraenoic acid), dexamethasone [DEX: (11β, 16α)-9-fluoro-11,17,21-trihydroxy-16-methylpregna-1,4-diene-3], naproxen [NAP: (S)-(+)-2-(6-methoxy-2-naphthyl)propionic acid], and esculetin (ESC: 6-hydroxy-7-methoxycoumarin) were purchased from Sigma-Aldrich Korea (Seoul, Korea) and dissolved in dimethyl sulfoxide (DMSO). Fura-8AM was purchased from AAT Bioquest (Sunnyvale, CA, USA) and dissolved in DMSO. Thromboxane B_2_ (TXB_2_: 9α,11,15S-trihydroxythromba-5Z,13E-dien-1-oic acid) and thromboxane A_2_ (TXA_2_: 9α,11α-methylene-15S-hydroxy-11α-deoxy-11α-methylene-thromba-5Z,13E-dien-1-oic acid) were purchased from Cayman Chemicals (Ann Arbor, MI, USA). DAN (dantrolene sodium: 1-[(5-(*p*-nitrophenyl) furfurylidene)amino] hydantoin sodium salt) were purchased from Sigma-Aldrich Seoul, Seoul, Korea. Prostaglandin E_2_ (PGE_2_: (5Z,11α,13E,15S)-11,15-dihydroxy-9-oxoprosta-5,13-dienoic acid), prostaglandin D_2_ (PGD_2_: 9α,15S-dihydroxy-11-oxoprosta-5Z,13E-dien-1-oic acid), prostaglandin I_2_ (PGI_2_: 6,9α-epoxy-11α,15S-dihydroxy-prosta-5Z,13E-dien-1-oic acid), 2-aminoethoxydiphenylborate (2-APB), thapsigargin (TPG), U-73122 (1-[6-[((17β)-3-methoxyestra-1,3,5[10]-trien-17-yl)amino]hexyl]-1H-pyrrole-2,5-dione), terutroban (TTB), and dazoxiben (DAZ) were purchased from Sigma-Aldrich Korea and dissolved in DMSO. Phosphate-buffered saline (PBS) was prepared with 100 mM phosphoric acid and adjusted to pH 7.4. Anticoagulant buffer (ACB) was prepared with 186 mM NaCl, 17 mM Na_2_EDTA, and 41 mM citric acid.

### Bioinformatics for Prediction of G Protein-Coupled Receptors

Based on GPCR sequences of *Bombyx mori* (GenBank accession number: NP_001037033.1)*, S. litura* (XP_022813850, XP_022814039, XP_022816105, XP_022817498, XP_022817499, XP_022821197, XP_022822728, XP_022822897, XP_022823006, XP_022826743, XP_022827237, XP_022827354, XP_022827490, XP_022827519, XP_022827626, XP_022834024, XP_022834058, XP_022834098, XP_022838045), *Heliothis virescens* (CDF56917.1), and *Trichoplusia ni* (XP_026733990), their orthologs of *S. exigua* were inferred from Transcriptome Shotgun Assembly (GGRZ01048721.1, GGRZ01057920.1, GGRZ01138956.1, GGRZ01102221.1, GGRZ01084708.1, GGRZ01092489.1, GGRZ01087211.1, GARL01043383.1, GGRZ01038487.1, GGRZ01037013.1, GARL01065745.1, GARL01040814.1, GGRZ01082913.1, GGRZ01068498.1, GGRZ01117535.1, GGRZ01069701.1, GGRZ01224538.1, GGRZ01247645.1, GGRZ01215736.1, GGRZ01095571.1, GGRZ01137765.1, GGRZ01106723.1, GGRZ01113408.1, GGRZ01069261.1) using the BlastN program (http://www.ncbi.nlm.nih.gov). The resulting sequences were subjected to open reading frame (ORF) analysis by using ORFfinder (https://www.ncbi.nlm.nih.gov/orffinder/). The ORF sequences were deposited to GenBank with accession numbers (AEO27700.1, AXY04240.1, AXY04245.1, AXY04246.1, AXY04251.1, AXY04254.1, AXY04275.1, AXY04297.1, AXY04299.1, AZA07970.1). Alternatively, the protein coding sequences of the GPCRs were inferred by mapping the protein sequences of the GPCR sequences at NCBI and newly identified ORF sequences against a reference whole genome assembly in *S. exigua* (GCA_011316535) using exonerate-2.2.0 with protein2genome model. Phylogenetic analysis was performed with the Neighbor-Joining method and a Poisson correction model using MEGA6.06 software (www.megasoftware.net). Bootstrapping values were obtained with 1,000 replications to test supports on each node in the resulting phylogenetic tree.

### Entomopathogenic Fungi Source and Culturing

Larvae of *S. exigua* infesting Welsh onion in Andong, Korea suffered from green muscardine disease symptoms and were collected for diagnosis. Fungal spores were collected in tubes containing sterile water by scraping from infected insect larvae. After stirring, 250 µL of the fungal suspension was spread on potato dextrose agar (PDA) and incubated at 25 ± 1°C and 70 ± 5% RH. The cultured spores were sub-cultured on PDA.

### Morphological Identification of EPF Isolate

The cultured fungal colonies were transferred onto slides with PVA mounting medium (PVA MTNG) (BioQuip Products, Gladwick Street, CA, USA) and incubated at 50°C for 48 h. The slides were observed under an optical microscope (DM500, Leica, Wetzlar, Germany) with 400× magnification. Fungal samples were collected from PDA cultured for 14 days and subjected to Au‐coating using a Sputter Coating machine (Jeol Korea, Seoul, Korea). The treated samples were observed under a scanning electron microscope (SEM; JSM‐6300, Jeol Korea) at 200 × magnification. The resulting morphological characters were used for the identification of the entomopathogenic fungi according to a taxonomy of key characters such as colony, conidiophore, and conidial shapes (29).

### Molecular Identification of EPF Isolate

Genomic DNA (gDNA) was extracted from a mixture of isolated spores and hyphae with phenol extraction and ethanol precipitation, as described by Park and Kim (17). Internal transcribed spacer (ITS) region was amplified using ITS forward primer (5’-TTGATTACGTCCCTGCCCTTT-3’) and ITS reverse primer (5’-TTTCACTCGCCGTTACTAAGG-3’) as reported by Vrain et al. (30). For PCR amplification of the ITS region, the extracted gDNA was used as a template with 35 cycles under the following conditions: 1 min at 94°C for denaturation, 1 min at 46°C for annealing, and 1 min at 72°C for extension. A second PCR was conducted using M13 universal primer-conjugated primers and sequenced by Macrogen (Seoul, Korea). The obtained nucleotide sequence was analyzed using the BlastN program of the National Center for Biotechnology Information (NCBI, www.ncbi.nlm.nih.gov). The evolutionary relationship was inferred using a Neighbor-Joining phylogenetic tree with MEGA6.06 (31). Bootstrap values on the branches were estimated with 1,000 replications.

### Pathogenicity of Fungal Isolate

A conidial suspension of *M*. *rileyi* was prepared by scraping the fungal culture into 1.5 mL tubes containing autoclaved Triton X-100 (0.1%) solution (Duksan Pure Chemicals, Ansan, Korea). After disentangling the conidial clumps by vortexing for 3 min, conidia in the suspension were counted using a Neubauer hemocytometer (Marienfeld-Superior, Lauda-Königshofen, Germany) under 40 × magnification.

The isolated fungi were fed to L3 larvae of *S. exigua* or *P. xylostella*, or 3.5 cm-length *T. molitor* larvae. A piece (1 × 1 cm) of cabbage containing 1,000 conidia was completely consumed by each larva. Each treatment was replicated 3 times and each replication consisted of 10 insects, in which each Petri dish held a single insect. The Petri dishes were kept in desiccators (4202-0000, Bel-Art Products, Pequannock, NJ, USA) maintaining a constant temperature of 25 ± 1°C and 75 ± 5% RH, following Winston and Bates (32) using a saturated solution of NaCl. Dead insects were counted every 24 h up to 7 days. For topical or injection application, 1,000 conidia per larva were used. Newly (< 30 min) molted fourth instar larvae were considered ‘unsclerotized’ while the larvae at 1 day post molting were considered ‘sclerotized’ cuticles. To remove the epicuticle layer of the integument, the abdominal tergum was rubbed with a brush soaked in acetone for 2 min.

### Ca^2+^ Signaling in Response to Fungal Challenge

To observe the Ca^2+^ signal and hemocyte aggregation behavior in response to fungal challenges (33), L5 larvae of *S. exigua* were injected with 2 µL Fura-8 (1 mM) and incubated for 30 min. 2 µL of fungal conidia and hyphae were injected. At selected time points post-injection (PI), hemolymph was collected and fixed on a slide glass by using 2.5% paraformaldehyde. The Fura-positive cells were observed under a fluorescence microscope (DM500, Leica, Wetzlar, Germany) at 400 × magnifications. Fluorescence change over time was analyzed using ImageJ software (https://imagej.nih.gov/ij). Each time point was replicated three times.

### Inhibition of Ca^2+^ Signaling

To inhibit Ca^2+^ flux, L5 larvae were co-injected with 1 µL of DAN (1 µg/µL), 2-APB (1 µg/µL), U-73122 (1 µg/µL), TPG (1 µg/µL), TTB (1 µg/µL), or DAZ (1 µg/µL) along with Fura-8 (1 mM). After 30 min, 2 µL of fungal conidia and hyphae were injected with TXB_2_ (1 µg). After another 10 min, hemolymph was bled and the hemocytes were fixed on a slide glass by using 2.5% paraformaldehyde. Aggregated hemocyte percentage and Fura-8-positive cells were determined as described above.

### Fluorescence Labeling of Conidia and Phagocytosis Assay

*M. rileyi* conidia were obtained from the 10-day culture on PDA medium. The conidia were resuspended in a sterile bicarbonate buffer (9.5 mL of 0.2 M Na_2_CO_3_ mixed with 41.5 mL of 0.2 M NaHCO_3_, pH 9.4). Then 1 µL of 10 mg/mL FITC fluorescein isothiocyanate (FITC; Sigma-Aldrich Korea) was added to the fungal pellet and placed on a shaker (170 rpm) for 30 min at room temperature under darkness (34). The conidia were washed four times with ice-cold PBS containing 0.02% EDTA and resuspended with 1 mL of TC100 insect medium (Welgen, Gyeongsan, Korea) and stored at -20°C.

For the phagocytosis assay, L5 larvae were surface-sterilized with 70% ethanol and injected with 2 µL of FITC-labeled conidia. At 30, 60, and 90 min PI, the larvae were bled through a clipped proleg onto a slide glass containing 5 µL of ACB. Phagocytic rates were determined by assessing the ratio of 100 hemocytes with or without ingested conidia under a fluorescence microscope (Leica, Wetzlar, Germany).

### Nodulation Assay

After surface sterilization with 70% ethanol, L5 larvae were anesthetized with ice and injected with 2 µL of conidial suspension (1×10^5^ conidia/mL) through the inter-segmental membrane between the last two abdominal segments using a Hamilton micro-syringe (Reno, NV, USA) equipped with a 26-gauge needle. After 8 h of incubation at room temperature, the hemocytic nodules were counted by dissecting the larvae under a stereomicroscope (Stemi SV 11, Zeiss, Jena, Germany) at 50 × magnifications. To assess the effects of DEX on nodule formation in larvae infected with *M. rileyi*, larvae were co-injected with 10 µg of DEX along with the conidia. For the time-course experiment, the number of nodules formed was counted at selected time points (1~8 h) PI, 3 larvae/time point.

### Hemocyte Aggregation Assay After Fungal Infection

Hemocyte aggregation is functionally defined as a clump of at least four hemocytes attached to conidia or hyphae. Aggregation activity was determined as the ratio of the aggregated hemocytes among 100 randomly chosen cells. To determine the inhibitory effects of eicosanoid biosynthesis inhibitors, each *S*. *exigua* L5 larva was injected with 1 µL of DEX (10 µg), ESC (1 µg), or NAP (1 µg) along with 2 µL of Fura-8 (1 mM). For the rescue experiment, 1 µL of AA (10 µg) was co-injected with DEX and 1 µL of PGE_2_ (1 µg) was co-injected with NAP. At 30 min PI, 2 µL of fungal conidia and hyphae were injected into individual insects. To determine the effect of eicosanoids on cellular immune reactions, individual *S. exigua* (L5) larvae were co-injected with 1 µL of DEX (10 µg) and 2 µL of Fura-8 (1 mM). At 30 min PI, the insects were injected again with 2 µL (1 µg) of prostaglandin D_2_, prostaglandin E_2_, prostaglandin I_2_, thromboxane A_2_, or thromboxane B_2_ along with fungal conidia and hyphae. At 10 min PI, hemolymph was collected and microscopic slides were prepared for assessing Fura-positive hemocyte and aggregated hemocytes.

### RNA Extraction, RT-PCR, and RT-qPCR

RNA extraction and cDNA preparation followed the method described by Vatanparast et al. (35). RT-PCR of *Se-TXAS* and *SePGE_2_R* genes was conducted using DNA Taq polymerase (GeneALL, Seoul, Korea) with an initial heat treatment at 94°C for 5 min, followed by 35 cycles of DNA denaturation at 94°C for 30 s, primer annealing at 50°C for 30 s (**Table S4**), and extension at 72°C for 30 s. The PCR reaction was completed with a final chain extension step at 72°C for 10 min. qPCR was conducted on a Real-time PCR thermal cycler (Step One Plus Real-Time PCR System, Applied Biosystems, Singapore) using Power SYBR Green PCR Master Mix (Life Technologies, Carlsbad, CA, USA) according to the guidelines of Bustin et al. (36). The reaction mixture (20 µL) contained 10 µL of PCR Master Mix, 5 µL sterile water, 3 µL of cDNA template (50 ng), and 1 µL each of forward and reverse primers (**Table S4**). The temperature program for qPCR began with 95°C heat treatment for 10 min followed by 40 cycles of denaturation at 94°C for 30 s, annealing at 50°C for 30 s, and extension at 72°C for 30 s. The expression level of the ribosomal gene, *L32*, was used as a reference gene to normalize target gene expression levels. Quantitative analysis was performed using the comparative CT (2^-ΔΔCT^) method (37).

### RNA Interference

For RNAi, double-stranded RNAs (dsRNAs) encoding *TXA_2_ synthase* (dsTXAS), *SePGE_2_R* (dsPGE_2_R), and *green fluorescence protein* (dsCON) were prepared as described by Vatanparast et al. (38) using Megascript RNAi Kit (Ambion, Austin, TX, USA). dsRNAs were mixed with a transfection reagent Metafectene PRO (Biontex, Plannegg, Germany) at a 1:1 (v/v) ratio and incubated at room temperature for 30 min to form liposomes. dsRNA (1 µg) was injected into L5 larval hemocoel with a microsyringe. The RNAi efficiency was evaluated by RT-qPCR at the selected time points. Each treatment was replicated three times using independent RNA preparations.

### PLA_2_ Activity Determination

Secretory PLA_2_ (sPLA_2_) activity in *S. exigua* larval plasma was determined at three different time points during incubation at 25°C using a commercial assay kit (sPLA_2_ Assay Kit, Cayman Chemical, Ann Arbor, MI, USA) with diheptanoyl thio-phosphatidyl choline as the enzyme substrate following Vatanparast et al. (38). Cellular PLA_2_ (cPLA_2_) activity measurement in fat body preparations used the same kit, with a different substrate, arachidonyl thio-phosphatidyl choline. A spectrofluorometer (VICTOR multi-label Plate reader, PerkinElmer, Waltham, MA, USA) was used to measure enzyme activity. Each treatment was replicated with three biologically independent enzyme preparations. Specific enzyme activity (µmol/min/µg) was calculated by dividing absorbance change by the amount of total protein. Protein concentrations were determined following Bradford (39).

### Total Hemocyte Count

Hemolymph was collected from L5 larvae into ACB by cutting an abdominal proleg and aspirating the exuded hemolymph with glass capillaries (TW100-4, World Precision Instrument, Sarasota, FL, USA). Hemocytes were counted with a hemocytometer (Neubauer improved bright line, Superior Marienfield, Lauda-Königshofen, Germany) under a phase contrast microscope (BX41, Olympus, Tokyo, Japan). Each treatment was independently replicated three times. To evaluate the effect of TXB_2_ on THC, test larvae were injected with TXB_2_ (1 µg per larva). Hemolymph was collected at each min after TXB_2_ treatment up to 10 min and assessed for THC as described.

### Effect of TXB_2_ on Hemocyte Micro-Aggregation

Micro-aggregation is defined as the aggregation of four or more hemocytes. L5 larvae were injected with different PGs and bled at different time points. The hemolymph (20 µL) was mixed with 10 µL of TC100 insect cell culture medium on a cavity well microscope slide (Globe Scientific, Mahwah, NJ, USA) to observe micro-aggregation behavior. Each treatment was replicated three times. To observe hemocyte migration on glass slides, 1 µL of TXB_2_ (1 µg) was added to 10 µL of hemocyte suspension obtained from naive larvae. The hemocyte behavior was monitored by fluorescence microscopy (Leica, Wetzlar, Germany) at 200×.

### Sample Preparation for TXB_2_ Analysis Using LC-MS/MS

Larvae immunity was challenged with 1,000 conidia injected into hemocoels of L5 larvae and incubated at 25°C for 16 h. Control larvae were injected with sterile PBS. Fat body samples were collected into 15 mL tubes from 70 larvae and washed with cold (4°C) PBS. Each sample was homogenized three times (10 min per cycle) in PBS with an ultrasonicator (Bandelin Sonoplus, Berlin, Germany) at 75% power, and subsequently adjusted to pH 4.0 using 1 N HCl. Prostanoids were extracted with 500 μL of ethyl acetate. The combined ethyl acetate extracts were dried under nitrogen to approximately 50 μL and applied to a small silicic acid column (2 × 90 mm containing 30 mg of Type 60A, 100-200 mesh silicic acid, Sigma-Aldrich Korea). Extracts were sequentially eluted with 300 μL of polar solvents starting with 100% ethyl acetate, followed by ethyl acetate: acetonitrile (1:1, v:v), 100% acetonitrile, acetonitrile: methanol (1:1, v:v), and 100% methanol. The acetonitrile:methanol fraction was used to quantify TXB_2_. Each treatment was replicated three times using independent sample collections.

### LC-MS/MS Analysis

LC-MS/MS was performed using a QTrap 4500 (AB Sciex, Framingham, MA, USA) equipped with an autosampler, a binary pump, and a column oven. The analytical column was an Osaka Soda C18 column (2.1 mm × 150 mm, 2.7 μm) maintained at 40°C (Osaka, Japan). The mobile phases consisted of 0.1% formic acid in water (A) and 0.1% formic acid in acetonitrile (B). The linear gradient was as follows: 30% B at 0 min, 30% B at 2 min, 65% B at 12 min, 95% B at 12.5 min, 95% B at 25.0 min, 30% B at 28.0 min, and 30% B at 30 min. The flow rate was 0.40 mL/min. The autosampler was set at 5°C and the injection volume was 10 μL. The LC-MS/MS was equipped with an electrospray ionization (ESI) source. ESI was performed in negative ion mode. After optimization, the source parameters were: source temperature at 400°C, curtain gas flow rate at 32 µL/min, ion source gas flow rate at 60 µL/min, and spray voltage adjusted to −4,500 V. Analyses were performed in Multiple Reaction Monitoring (MRM) detection mode using nitrogen as the collision gas. Peak detection, integration, and quantitative analysis were done using MassView1.1 software (AB Sciex).

### Statistical Analysis

The data for continuous dependent variables were subjected to one-way analysis of variance (ANOVA) using PROG GLM in the SAS program (40). Mortality assay by the leaf dipping method was analyzed by repeated measure ANOVA. All experiments were conducted in three biologically independent replicates and the means ± standard errors (SE) were plotted using Sigma Plot (Systat Software, Point Richmond, CA, USA). The means were compared with the least significant difference (LSD) test at a Type I error of 0.05.

## Results

### Identification and Pathogenicity of Fungal Isolates

Fungal spore colonies on the PDA medium began to grow slowly with a white velvety appearance (early stage) and irregular borders at 4 days, then turned to pale green or malachite green with sporulation at 7 days (**Figure S1A**). The vegetative and reproductive structures developed hyaline hyphae and conidiophores with smooth and septate walls. The branches, which formed near the septa, developed in clusters on the same point with 2~4 phalids exhibiting a short, rounded, and thickened base. Conidia were smooth and ellipsoidal, and arranged in chains.

The ITS region was amplified and sequenced to identify species in the fungal isolate (**Figure S1B**). In total, 734 bp were sequenced, including full-length 5.8S rRNA, ITS-1, and ITS-2 regions as well as partial sequences of 18S and 28S rRNAs. The sequences showed high similarities (>99%) with known ITS sequences of several *M*. *rileyi* strains (**Table S1**). To evaluate the relationship of the isolate with other fungal strains, a phylogenetic tree was constructed using the ITS sequences (**Figure S1C**). The isolate was significantly (bootstrap value = 100%) clustered with another *M*. *rileyi* isolate.

The fungal isolate was pathogenic to tested insects, with considerable variation in the fungal virulence (**Table S2**). The lethal median time (LT_50_) was much faster against *S. exigua* (111.41 h) than *T*. *molitor* (182.65 h) or *P*. *xylostella* (136.35 h). The insecticidal activities of the fungal isolate were also more potent in *S. exigua* (76.7%) compared to other insects under the same fungal treatment at 7 days after treatment (**Figure S1D**).

### Eicosanoids Mediate Immune Responses to *M. rileyi* Infection

**Figure 1:**
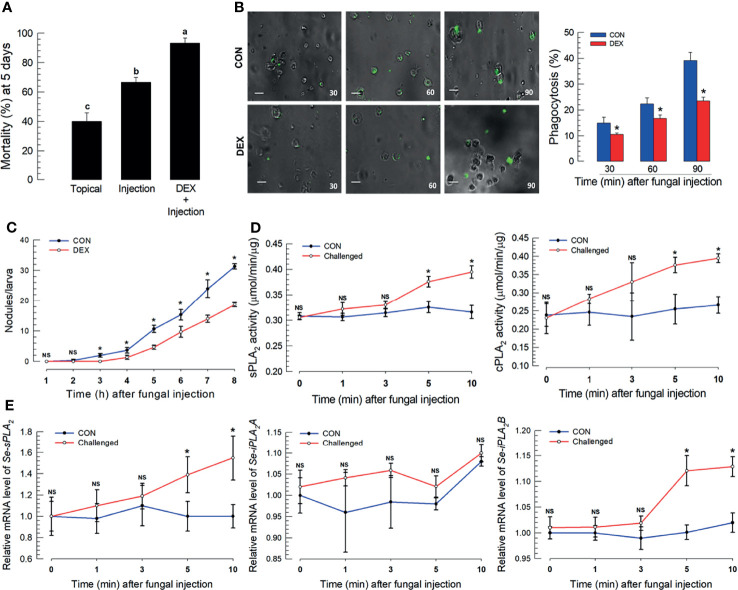
*S. exigua* immune responses to a *M. rileyi* fungal infection. For all panels, control (‘CON’) represents solvent (DMSO) injection. Panel **(A)** shows the influence of fungal topical application or injection (1,000 conidia per L5 larva) on mortality at 5 DPF. Co-injections of DEX (10 μg/larva)+conidia led to increased mortality. Each treatment was replicated three times with 10 larvae per replication. Panel **(B)** reports the influence of DEX on phagocytosis, determined by fluorescence emitted from internalized FITC-labeled conidia. The assays were replicated at the indicated time points with biologically independent samples. Scale bar represents 10 μm. The accompanying histogram shows proportions of phagocytic hemocytes at the indicated times. Panel **(C)** shows mean nodules/larvae at the indicated times PFI. L5 larvae were injected with 2 µL of conidia (1×10^5^ conidia/mL). The number of nodules were counted in three larvae at each time point. Panel **(D)** shows sPLA_2_ activities in plasma and cPLA_2_ activities in fat body at the indicated times PFI (n = 3 biologically independent replicates with 10 larvae/replicate). Panel **(E)** exhibits relative accumulations of mRNAs encoding the indicated PLA_2_s at 1, 3, 5, and 10 h PFI. ‘NS’, no significant difference. Data analyzed and presented as described in *Methods*. Different letters or asterisks above standard deviation bars indicate significant difference among means at Type I error = 0.05 (LSD test).

Fungal virulence to *S. exigua* larvae differed by infection route. Topical application led to 40.0% mortality, which increased to 64% following spore injections (**Figure 1A**). *M. rileyi* virulence increased in newly molted larvae, before cuticular sclerotization (**Figure S2A**) or to integument treated with an organic solvent to remove the epicuticular layer (**Figure S2B**). Co-injections of spores with DEX led to substantially increased mortality (**Figure 1A**). The DEX treatment led to significantly suppressed phagocytosis (**Figure 1B**) and hemocyte nodule formation following fungal infection (**Figure 1C**). sPLA_2_ and iPLA_2_ activities significantly increased within 5 min post-fungal infection (PFI; **Figure 1D**), with parallel increases in mRNAs encoding sPLA_2_ and iPLA_2_B, but not iPLA_2_A (**Figure 1E**).

### Eicosanoids Induce Intracellular Ca^2+^ Mobilization

**Figure 2:**
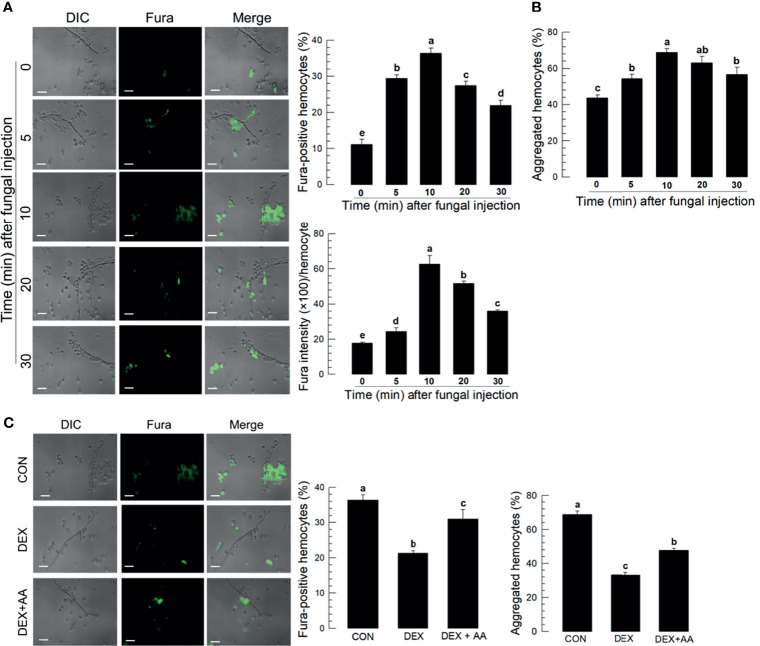
Ca^2+^ signal in aggregating hemocytes to fungal hyphae of *M. rileyi* in *S. exigua*. Panel **(A)** conveys the induction of Ca^2+^ signals in *S. exigua* hemocytes. Larvae were injected with 1,000 conidia/L5 larva at 30 min after injecting 2 µL Fura, 1 mM). At the indicated time points, hemolymph was collected and fixed with 2.5% paraformaldehyde. Fluorescent hemocytes were counted and changes in fluorescence intensity were recorded as described in *Methods*. Panel **(B)** shows proportions of Fura-positive hemocytes (upper left), proportions of aggregated hemocytes (upper right), and Fura intensities/100 hemoytes at the indication times PFI. Panel **(C)** presents the influence of DEX (10 μg/larva) on hemocyte Ca^2+^ signals and hemocyte aggregating behavior after fungal infection. The left panel presents visual images hemocytes after the indicated treatments. The middle histogram reports proportions of Fura-positive hemocytes in controls, hemocytes after DEX exposure, and hemocytes after DEX+AA (10 μg/larva). Each treatment was replicated three times. Data analyzed and presented as described in Methods. Scale bar represents 10 μm. ‘DIC’ represents differential interference contrast. ‘CON’ represents solvent (DMSO) control. Different letters above standard deviation bars indicate significant difference among means at Type I error = 0.05 (LSD test).

Hemolymph collected from *M. rileyi*-challenged larvae had labeled spores and hyphae connected to hemocytes (**Figure 2A**). Proportions of Fura-positive hemocytes increased with time, up to 10 min PFI, in correlation (*r* = 0.943; *P* < 0.0001) with numbers of hemocyte aggregates (**Figure 2B**). Fura-staining intensity also increased with time up to 10 min. Treating *S. exigua* larvae with the PLA_2_ inhibitor, DEX, led to reduced proportions of Fura-positive hemocytes and aggregated hemocytes (**Figure 2C**).

### Prostanoids Influence Ca^2+^ Mobilization and Hemocyte Microaggregation

**Figure 3:**
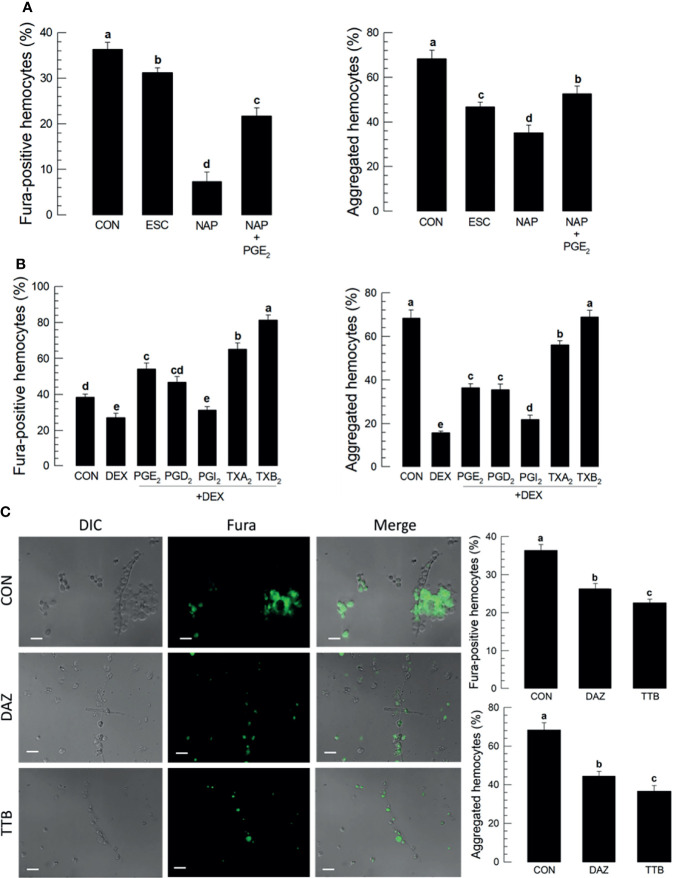
Thromboxanes (TXA_2_ and TXB_2_) induce Ca^2+^ signaling and hemocyte aggregation in *S. exigua*. Panel **(A)** reports that the COX inhibitor, NAP, but not LOX inhibitor, ESC, led to reduced proportions of Fura-positive hemocytes and reduced proportions of aggregated hemocytes. Co-injections with NAP+PGE_2_ (1 µg/µL) rescued the influence of inhibiting COX. Panel **(B)** displays the influence of DEX on Ca^2+^ signaling and hemocyte aggregation, which were reversed in larvae treated with DEX+the indicated prostanoids. Panel **(C)** shows the influence of the thromboxane biosynthesis inhibitor DAZ and the thromboxane receptor antagonist, TTB, on Ca^2+^ signaling and hemocyte aggregation. The micrographs convey visual images, with reduced Fura signaling and the accompanying histograms show quantitative data with statistical analysis. Each treatment was replicated three times. Data analyzed and presented as described in *Methods*. Different letters above standard deviation bars indicate significant difference among means at Type I error = 0.05 (LSD test).

Treating larvae with a nonspecific cyclooxygenase inhibitor, NAP, prior to the conidial challenge led to substantially suppressed proportions of Fura-positive hemocytes (down by about 40%) and hemocyte microaggregates (down by about 35%), both of which were reversed in larvae co-treated with NAP+PGE_2_ (**Figure 3A**). Treatment with the lipoxygenase inhibitor ESC led to small but statistically significant reductions in both parameters, with Fura-positive proportions down by about 8% and aggregated hemocytes down by about 24%. Treating larvae with DEX led to reductions in proportions of Fura-positive hemocytes (down by about 20%) and aggregated hemocytes (down by about 50%). **Figure 3B** shows the influences of selected prostanoids on possible reversals of DEX treatments before the conidial challenge. Co-injecting DEX along with one of three PGs led to increases in both parameters (**Figures S3A–E**). PGE_2_ treatment led to increased Fura-positive hemocyte proportions by about 56%; PGD_2_ treatment increased by about 50%; PGI_2_ treatment did not reverse the DEX effect. TXA_2_. TXB_2_ treatments led to very high proportions of Fura-positive hemocytes, up to just over 60% for TXA_2_ and nearly 80% for TXB_2_. Parallel experiments with proportions of aggregated hemocytes led to similar results. **Figure 3C** shows the outcomes of treating larvae with specific inhibitors of thromboxane synthesis, DAZ, and a thromboxane receptor antagonist, TTB. Compared to controls, co-injecting conidia+DAZ and, in a separate group of experimental larvae, conidia+TTB led to significant decreases in proportions of Fura-positive hemocytes (down by about 10% after DAZ treatment and by about 20% after TTB treatment) and aggregated hemocytes (down by about 20% after DAZ treatment and by about 35% after TTB treatment).

**Figure 4:**
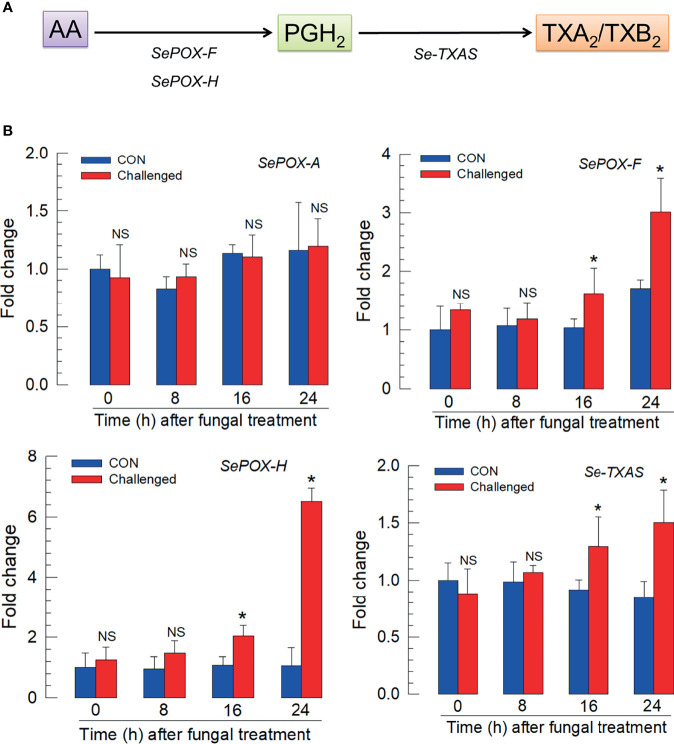
Up-regulation of thromboxane biosynthesis in *S. exigua* hemocytes following *M. rileyi* infection. Panel **(A)** presents a likely thromboxane biosynthetic pathway. AA is oxygenated to PGH_2_ by two peroxinectins, SePOX-F’ and SePOX-H. Thromboxane A_2_ (‘TXA_2_’) is then formed by Se-TXAS and non-enzymatically converted to thromboxane B_2_ (‘TXB_2_’). Panel **(B)** reports the influence of *M. rileyi* infection on accumulations of mRNAs encoding SePOX-A, SePOX-F, SePOX-H, and Se-TXAS. Each treatment was replicated three times. Data analyzed and presented as described in *Methods*. ‘NS’, no significant difference. Asterisks above standard deviation bars indicate significant difference among means at Type I error = 0.05 (LSD test).

**Figure 4A** outlines the biosynthesis of TXA_2_/TXB_2_ from AA in *S. exigua*. Two POXs, SePOX-F and SePOX-H, convert AA into PGH_2_, which is converted into TXA_2_. TXA_2_ can convert into TXB_2_ spontaneously. **Figure 4B** shows that the *M*. *rileyi* challenge did not influence the time-dependent accumulation of mRNAs encoding *SePOX-A* (upper left), while accumulations of mRNAs encoding *SePOX-F* and *SePOX-H* were significantly elevated beginning 16 h PFI (upper right and lower left panels). **Figure 4B** (lower right) shows that the accumulation of mRNAs encoding *Se-TXAS*, the enzyme that converts AA into TXA_2_, was also significantly elevated at 16 h PFI.

**Figure 5:**
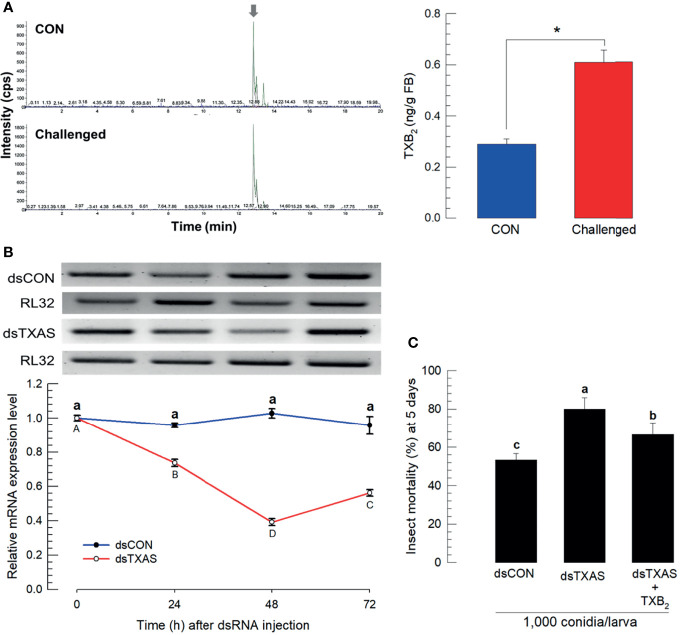
Mass spectral determination of TXB_2_ and influence of silencing *SeTXAS* on mortality. Panel **(A)** shows that the fungal challenge led to increased concentrations of larval fat body TXB_2_ at 16 h PFI. An arrow shows TXB2 peaks on LC-MS chromatograms Panel **(B)** shows the influence of injecting an RNAi construct designed to *SeTXAS* on accumulations of SeTXAS mRNAs. Control dsRNA (‘dsCON’) used dsRNA specific to *GFP*. Panel **(C)** shows the influence of dsTXAS on L4 larval mortality at 5 days PFI. Each treatment was replicated three times. Data analyzed and presented as described in *Methods*. Different letters or asterisks above standard deviation bars indicate significant difference among means at Type I error = 0.05 (LSD test).

We estimated concentrations of TXB_2_ in fat body (L5 larvae) by LC-MS/MS. Chemical identification of TXB_2_ was confirmed by two specific ion peaks, which matched the ion peaks in an authentic TXB_2_ chemical standard (**Figure S4**). TXB_2_ was detected at 0.28 ± 0.02 ng/g in naïve larvae, which was significantly increased to 0.61 ± 0.05 ng/g in *M*. *rileyi*-challenged larvae (**Figure 5A**). *Se-TXAS* expression was silenced by injecting dsRNA specific to the gene into experimental larvae (**Figure 5B**). Injecting *M*. *rileyi* into dsRNA-treated larvae led to significantly enhanced pathogenicity, which was reversed in larvae co-injected with conidia+TXB_2_ (**Figure 5C**).

### The Influence of TXB_2_ on Hemocyte Behavior

**Figure 6:**
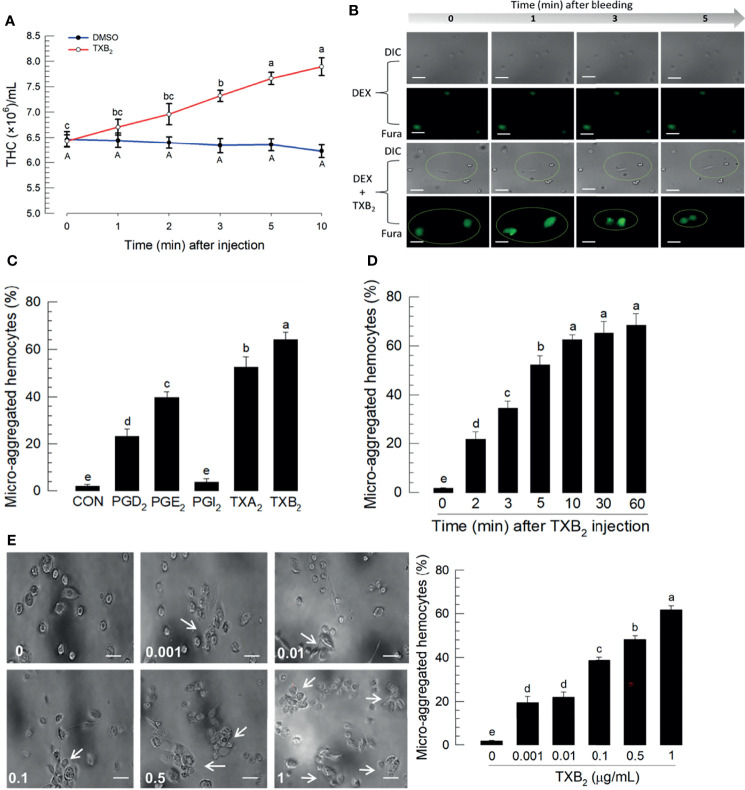
Influence of time on hemocytic immunity in *S. exigua*. Panel **(A)** depicts time-dependent up-regulation of total hemocyte count (THC) in L5 larvae after TXB_2_ injection at 1.0 µg/larva. Panel **(B)** displays hemocyte migration activated by TXB_2_ as a function of time after hemolymph collection, inhibited in DEX-treated larvae, and rescued in DEX+TXB_2_-treated larvae. Dotted circles indicate hemocyte migration. Panel **(C)** shows the influence of PGD_2_, PGE_2,_ and the two thromboxanes on hemocyte microaggregation. Panel **(D)** depicts the time-dependent hemocyte microaggregation following larval TXB_2_ injection 1 µg/mL in *in vitro* hemocyte preparations. Panel **(E)** shows dose response of the hemocyte migration 10 min after TXB_2_ treatment in *in vitro* hemocyte preparations from naïve larvae. The white arrows point to microaggregates. ‘DIC’ represents differential interference contrast. Each treatment was replicated three times. Data analyzed and presented as described in *Methods*. Different letters above standard deviation bars indicate significant difference among means at Type I error = 0.05 (LSD test).

**Figure 6A** reports the influence of TXB_2_ injections (1 μg/larva) on total hemocyte numbers over 10 h PI. The blue line represents DMSO-treated control larvae and the red line shows total hemocyte counts increase over time PI. **Figure 6B** shows reports that DEX injections led to no visible changes in DIC, nor Ca^2+^ signaling. Co-injecting DEX+TXB_2_ visible DIC and Fura staining. **Figure 6C** shows that separate treatments with the indicated prostanoids led to substantial increases in proportions of microaggregated hemocytes with the exception of PGI_2_ treatments, which did not influence microaggregation. The thromboxane injections led to statistically significant higher proportions of microaggregated hemocytes, compared to controls and the three PG treatments. **Figure 6D** reports proportions of microaggregated hemocytes steadily increase as a function of time over 60 min after TXB_2_ injections to a maximal level at about 60% of total hemocytes. This stimulatory activity of TXB_2_ exhibited a dose-dependency (**Figure 6E**).

### Thromboxanes Influence Hemocyte Behavior *via* a Specific Receptor

**Figure 7:**
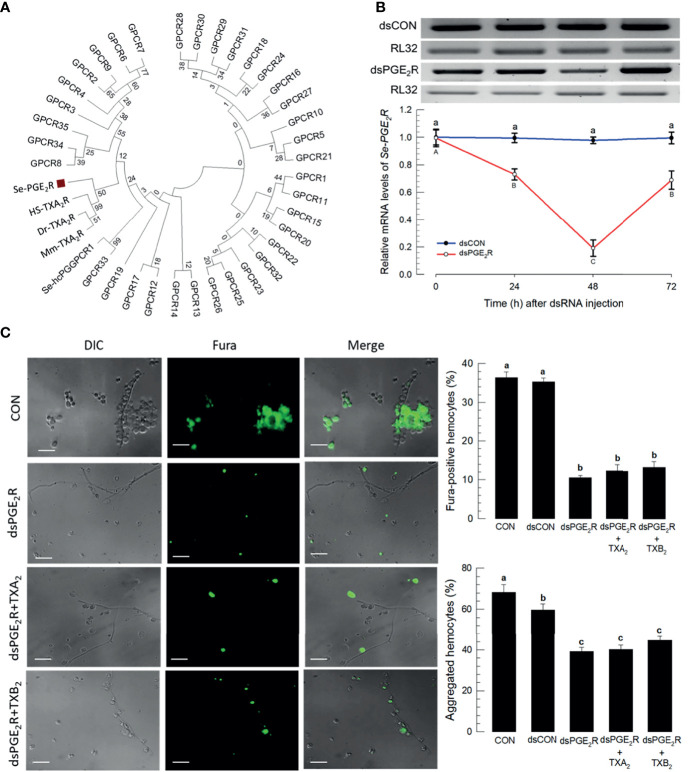
A PGE_2_ receptor mediates thromboxane signaling in S*. exigua*. Panel **(A)** shows a phylogenetic analysis of 35 *S. exigua* G-protein coupled receptors (GPCRs) indicating two PGE_2_ receptors (‘Se-PGE_2_R’ and Se-hcPGGPCR1’). The Se receptors are closely aligned with human (‘Hs’), mouse (‘Mm’), and fish (‘Dr’) TXA_2_ receptors (‘TXA_2_R’) using the Neighbor-Joining method with MEGA6.06. GenBank accession numbers of these genes are presented in Supplementary data (**Table S4**). Bootstrap values on the branches were estimated with 1,000 repetitions. The black square indicates a clustering with vertebrate TXA_2_Rs. Panel **(B)** indicates the influence of a dsPGE2R construct on accumulations of mRNAs encoding Se-PGE_2_R as a function of time post-injections. Control dsRNA (‘dsCON’) used dsRNA specific to *GFP*. Panel **(C)** shows the influence of dsPGE2R on Ca^2+^ signaling and hemocyte aggregation, which was not reversed after co-injection of dsPGE2R+TXA_2_ and dsPGE2R+TXB_2_. ‘DIC’ represents differential interference contrast. ‘CON’ represents the fungal infection alone. Each treatment was replicated three times. Data analyzed and presented as described in *Methods*. Different letters above standard deviation bars indicate significant difference among means at Type I error = 0.05 (LSD test).

Within the *S. exigua* genome there are 37 predicted GPCR candidates (**Table S3**), which were used to be compared with mammalian PG receptors. In particular, **Figure 7A** shows that a *S. exigua* PGE_2_ receptor (Se-PGE_2_R) is clustered with human, house mouse, and zebrafish thromboxane receptors. Injecting a dsRNA construct designed to the SePGE_2_R sequence led to severe reductions in mRNAs encoding *Se-PGE_2_R*, down by about 80% (**Figure 7B**). **Figure 7C** shows that silencing Se-PGE_2_R led to reduced accumulations of mRNAs encoding PGE_2_R and reduced Ca^2+^ signaling, seen in microphotographs as severe reductions in Fura-positive hemocytes and the accompanying histogram. Co-injections of dsPGE_2_R+TXA_2_ and, separately, dsPGE_2_R+TXB_2_, did not restore FURA staining. Similar images and the accompanying histogram document the same outcomes with proportions of microaggregated hemocytes.

### Thromboxane Up-regulates Ca^2+^ *via* Ca^2+^-Induced Ca^2+^ Release Pathway

**Figure 8:**
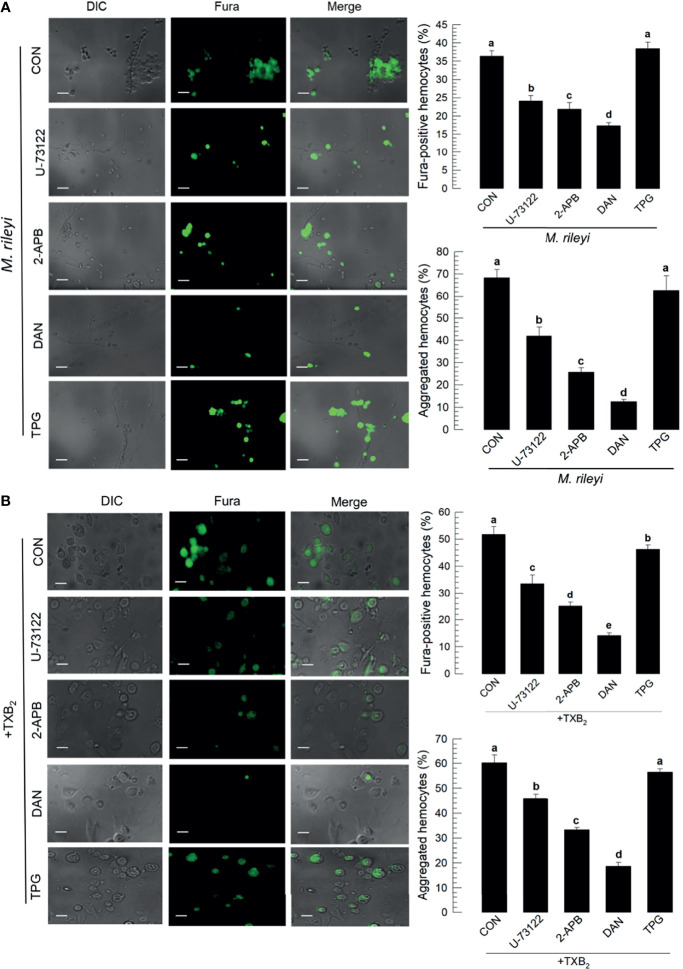
Influence of Ca^2+^ signaling inhibitors on Ca^2+^ signaling in *S. exigua* hemocytes. Panel **(A)** depicts the influences of separate treatments with DAN, a ryanodine receptor inhibitor, 2-APB an IP_3_ receptor inhibitor, U-73122, a PLC inhibitor, and TPG, a SERCA inhibitor on proportions of Fura-positive hemocytes and proportions of aggregated hemocytes following *M. rileyi* injections. The micrographs show Fura-positive hemocytes and the accompanying histograms present the results in quantitative terms. Panel **(B)** indicates the negative influence of the Ca^2+^ signaling inhibitors was not rescued by TXB_2_ treatments. CON represents the fungal infection or TXB_2_ alone. Each treatment was replicated three times. Data analyzed and presented as described in *Methods*. Different letters above standard deviation bars indicate significant difference among means at Type I error = 0.05 (LSD test).

The signaling pathway to up-regulate Ca^2+^ signal in hemocytes in response to TXB_2_ was monitored by treating larvae with selected compounds that influence Ca^2+^ signaling in mammalian cells (**Figure 8**). The micrographs in **Figure 8A** show that phospholipase C inhibitor (‘U-73122’), IP_3_ receptor inhibitor (2-APB), and ryanodine receptor inhibitor (‘DAN’) significantly suppressed Ca^2+^ signaling and hemocyte aggregation following the *M*. *rileyi* challenge. The accompanying histograms show the quantitative values. In contrast, a sarco/endoplasmic reticulum Ca^2+^-ATPase (SERCA) inhibitor (‘TPG’) did not suppress the Ca^2+^ signal. The micrographs in **Figure 8B** and their accompanying histograms similarly show that co-injections with TXB_2_ did not reverse the influences of the drug treatments.

## Discussion

Entomopathogenic fungi are effective biological control agents deployed in a range of ecological settings and they are used in bioremediating environmental toxins (41). Their market values are rapidly growing (42), in parallel with their use. Nonetheless, the understanding insect physiological and molecular mechanisms of host defense against invading fungi remains incomplete. Here, we contribute a new understanding of the biochemical signaling mechanisms responsible for host cellular immune responses to infections by an entomopathogen, *M. rileyi*, which is known to be a pathogenic fungus to *S. exigua* (17). Specifically, we identified two eicosanoids, the prostanoids TXA_2_ and TXB_2_, as key signals responsible for hemocyte migration to infection foci, phagocytosis of fungal conidia, and hemocyte microaggregation reactions to infection.

The entomopathogenicity of *M. rileyi* was enhanced by the addition of DEX, a pharmaceutical PLA_2_ inhibitor. PLA_2_ is the first step in the biosynthesis of all eicosanoids and inhibiting this enzyme effectively eliminates eicosanoid signaling. Here, DEX treatments led to increased mortality and decreased phagocytosis, nodulation, hemocyte aggregation, activity of sPLA_2_ and cPLA_2_, and intracellular Ca^2+^ signaling. We infer eicosanoids signal all these activities, which is supported by the outcomes of injecting AA with DEX, which reversed the reductions. *M. rileyi* infections led to increases in several related parameters. The fungal challenge led to increases in sPLA_2_ and cPLA_2_ activity and increases in accumulations of mRNAs encoding several eicosanoid-related enzymes, Se-sPLA_2_, Se-iPLA_2_A, Se-iPLA_2_B, SePOX-F, SePOX-H (but not SePOX-A), and SeTXAS. We infer eicosanoids, particularly TXA_2_ and TXB_2_, are central actors in the immune response to fungal infections.

Among different eicosanoids, PGs mediated the Ca^2+^ signal in response to the fungal infection. Inhibiting the lipoxygenase pathways with ESC treatments did not influence Ca^2+^ signaling nor hemocyte aggregation, while separate experiments with the cyclooxygenase inhibitor naproxen (NAP) led to sharp reductions in both parameters, with Ca^2+^ signaling down by about 30% and aggregation down by about 45%. The reductions were reversed following co-injection of NAP+PGE_2_. Hence, PGs, but not lipoxygenase products, operate in the two hemocyte defense parameters. We found that separate co-injections of DEX + two PGs, PGE_2_ and PGD_2_, but not DEX+PGI_2_, reversed the DEX inhibitory effects on Ca^2+^ signaling and hemocyte aggregation, to some extent. DEX+TXA_2_ and DEX+TXB_2_ treatments led to steep increases in Ca^2+^ signaling, higher than controls, while separate DEX+TXB_2_ co-injections returned hemocyte aggregations to control levels. Experiments with two thromboxane-specific compounds, DAZ and TTB, emphasize the point. DAZ specifically inhibits TXB biosynthesis and TTB is a TBX receptor antagonist. Treatments with these compounds led to significantly reduced Ca^2+^ signaling and hemocyte aggregation. Our interpretation is that prostanoids, particularly the two thromboxanes, mediate these hemocytic reactions to fungal infections.

Physiological activities take place over time and analysis of the influence of time reveals subtle aspects of thromboxane signaling in insect responses to the fungal challenge. Fungal infections led to increased phagocytosis, recorded as about 15% at 30 min PFI, which increased to 22% by 60 min, and approximately doubled over the next hour. We infer that phagocytosis accelerated over the 3 h time frame. Nodulation reactions to infection were undetectable until 3 h PFI, then increased from about 2 nodules/larva to nearly 30/larvae over the next 6 h, increasing in a non-linear manner. Accumulation of mRNAs encoding PLA_2_ took place on a different time-scale, with increases recorded from 3 to 10 min PFI. Two cellular parameters, Ca^2+^ signaling (recorded as changes in Fura intensities) and aggregated hemocytes, increased over the first 10 min PFI, then declined significantly over the next 20 min. This is also apparent in micrographs taken over the same time frames. We note, also, that hemocyte microaggregation proportions steadily increased with time for 60 min PI. Our broad point is that insect immune reactions take place over time, and our appreciation of the time dimension enriches our understanding of immunity.

Research with eicosanoids is challenging because these compounds are produced and operate in very small amounts. Physiological quantities of PGs and other eicosanoids are often determined indirectly using radioimmunoassays and bioassays. Sensitive mass spectrometry now enables accurate direct determinations of eicosanoid quantities at physiological levels. Our data show TXB_2_ occurred in the fat body of naïve larvae at approximately 0.28 ng/g, which increased by 2.2-fold to about 0.61 ng/g at 16 h PFI. This chemical determination strongly bolsters our view that TBX_2_ is present and operates in larval tissues. Injecting a dsRNA construct specific to *SeTXAS* led to reduced accumulations of mRNAs encoding *SeTXAS* at 24, 48, and 72 h PI. Treatments with the gene silencing construct led to increased larval mortality at 5 days PFI, which was significantly, albeit not completely, reversed in larvae co-injected with dsTXAS+TXB_2_.

One eicosanoid action in insect cellular immunity is the activation of Ca^2+^ signaling within hemocytes in mealworms, *Tenebrio molitor*. Roy and Kim (33) reported that bacterial infections stimulate Ca^2+^ activation, recorded as Fura-positive cells. One role of Ca^2+^ is activating hemocyte spread by F-actin extension, documented by co-localization of Ca^2+^ and F-actin. In this report, we consider Ca^2+^ mobilization in more detail by applying selected pharmaceuticals. U-73122 is a phospholipase C inhibitor that inhibits Ca^2+^ release from endoplasmic reticulum (ER) stores (43). 2-APB influences a wide range of channels, including Ca^2+^ channels, possibly in an indirect manner by cytoplasmic acidification (44). DAN inhibits ryanodine receptors (45), a class of intracellular Ca^2+^ channels located in ER and responsible for releasing Ca^2+^ from intracellular stores (46). SERCA operates in Ca^2+^ uptake by transferring Ca^2+^ from the cell cytoplasm into the lumen of the sarcoplasmic reticulum or endoplasmic reticulum. TPG treatments did not influence intracellular Ca^2+^ signaling, nor hemocyte aggregation.

Upon fungal infection, hemocytes physically attach to the fungal hyphae or conidia. The hemocytes aggregating around the fungi exhibited intensive Ca^2+^ signaling The increased Ca^2+^ signal was positively associated with hemocyte aggregation behavior. However, DEX treatment suppressed the Ca^2+^ signal and inhibited hemocyte aggregation. This inhibition was rescued by adding AA. We infer that eicosanoids induce Ca^2+^ signaling to activate hemocyte aggregation. Ca^2+^ is required for hemocyte behavior. In *M. sexta*, plasmatocytes require Ca^2+^ to facilitate spreading (47). Indeed, an endoparasitoid wasp against *D. melanogaster* encodes a Ca^2+^ blocker mimicking SERCA to shut down Ca^2+^ bursts, which results in the host immunosuppression (48).

Among eicosanoids, thromboxane treatments (TXA_2_ and TXB_2_) highly activated the intracellular Ca^2+^ signal, which led to hemocyte aggregation in response to fungal infection. Among other PGs known in *S. exigua*, PGD_2_ and PGE_2_ also activated the hemocyte behavior while PGI_2_ did not because it acts as an anti-inflammatory mediator (49). TXA_2_ mediates blood clotting in mammals by reducing blood flow to the site of a clot through vasoconstriction and by aggregating platelets to the site (13). In insects, the wound healing process along with coagulation factors is mediated by eicosanoids, shown by using a PLA_2_-mutant line of *Drosophila* (15). This study shows increased TXB_2_ titer in response to the fungal infection. We also observed that the total hemocyte numbers in the hemolymph and their migratory behavior were increased by TXB_2_. We infer that thromboxanes contribute to cellular immune responses, which include the wound healing process by stimulating hemocyte aggregation to the infection foci.

Terutroban has been used to inhibit the mammalian thromboxane receptor (TP) (50). It inhibits TXA_2_ or TXB_2_ action to mediate the Ca^2+^ signal and hemocyte aggregation in *S. exigua*. This suggested that the thromboxane actions are mediated through TP-like receptors in insects. In humans, TP receptors exist in two alternative splicing variants, TPα and TPβ, in which TPα is the dominant isoform translated in platelets and vascular cells, and the TPβ isoform is present in vascular smooth muscle cells (51, 52). TPs are classified into seven transmembrane GPCRs. Despite no TP ortholog in *S. exigua* genome, a PG receptor, *Se-PGE_2_R*, is required for TXB_2_ to mediate the Ca^2+^ signal and immune responses. Like mammals, insects produce three groups of prostanoids, PGs, prostacyclin, and thromboxane (22, 26, 49). Although different prostanoids act *via* specific GPCRs in mammals, there are multiple receptors, cross-reactivity, and cross-talks for each prostanoid, in which PGE_2_ is the most versatile prostanoid because of four different receptor subtypes (53). Two PG receptors were known in *S. exigua*. The first PGE_2_ is specific to oenocytoid hemocytes and induces the hemocyte cell lysis to release prophenoloxidase for melanization during cellular immune responses (54). The second receptor (*Se-hcPGGPCR1*) is expressed in various cell types and mediates immune and reproductive processes (26). The latter *SePGE_2_R* is closely associated with the mammalian TPs in phylogenetic analysis, and its RNAi treatment prevented the immunological functions of thromboxanes in *S. exigua*. These suggest that SePGE_2_R is shared by two prostanoids. This kind of receptor-ligand interaction is explained by a functional pleiotropy in PRXamide neuropeptides and their receptors, exhibiting differential binding affinities (55). Based on this pleiotropism, differential binding affinities of PGE_2_ and TXB_2_ to common PG receptors may form orchestrated multi-organ physiological outcomes. This hypothesis will be tested through receptor-ligand binding assays.

**Figure 9:**
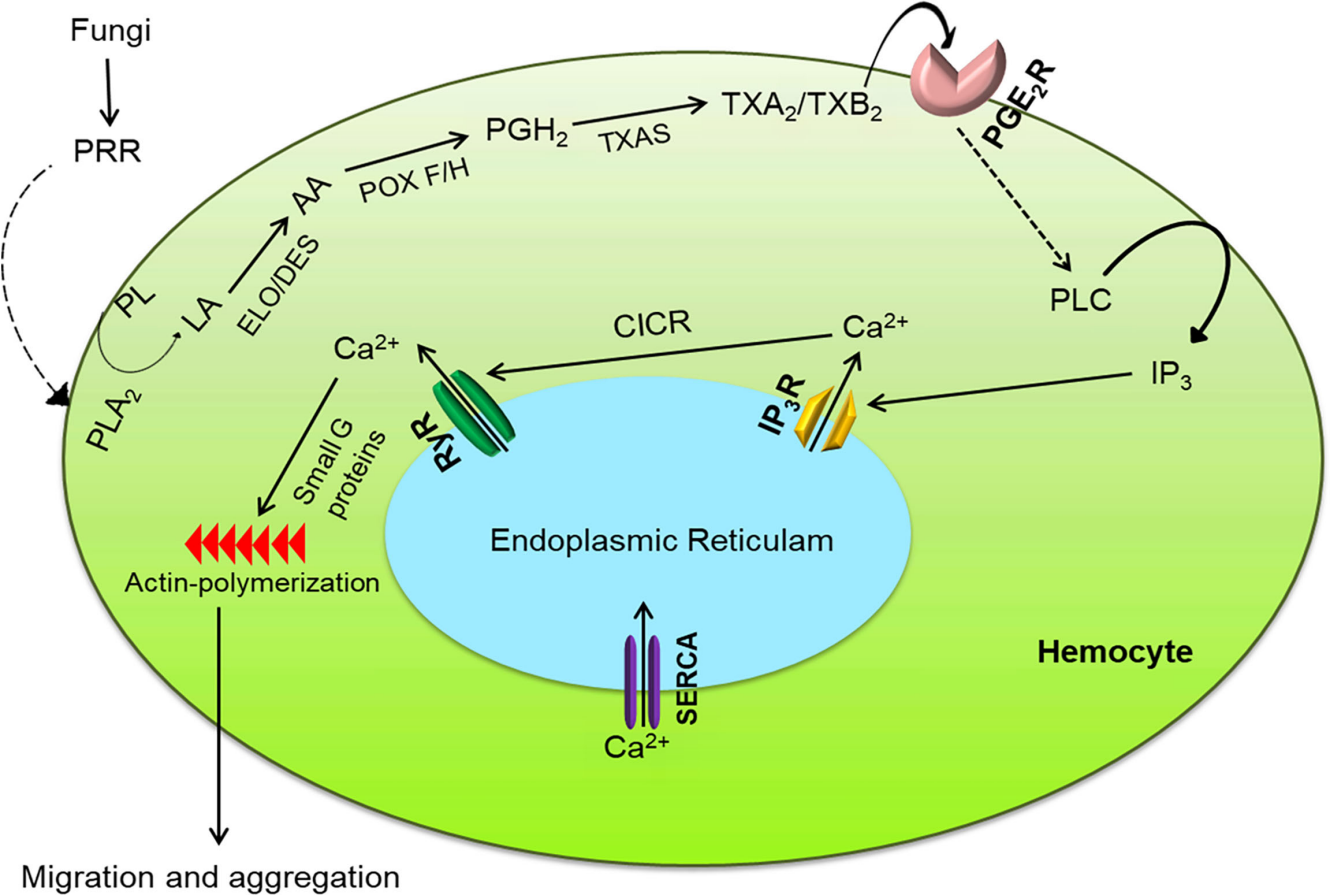
Intracellular immune signaling in *S. exigua* hemocytes following fungal infection. Fungal hyphae or conidia are recognized by pattern recognition receptor (‘PRR’), which activates the Toll pathway to increase PLA_2_ activity. The activated PLA_2_ releases linoleic acid (‘LA’), which is desaturated and elongated into arachidonic acid (‘AA’). AA is then oxygenated by two peroxinectins (‘POX F/H’) into PGH_2_, which is isomerized into TXA_2_/TXB_2_ by TXA_2_ synthase (‘TXAS’). TXA_2_/TXB_2_ is transferred out of the cell to bind with its GPCR (‘PGE_2_R’) and activate phospholipase C (‘PLC’). PLC increases intracellular inositol triphosphate (‘IP_3_’) concentrations which binds to its receptor (‘IP_3_R’) on endoplasmic reticulum, thereby releasing Ca^2+^ into the cytoplasm Ca^2+^ triggers calcium-induced calcium release (‘CICR’) from a ryanodine receptor (‘RyR’). The free Ca^2+^ is a secondary signal to activate small G proteins for actin polymerization to facilitate hemocyte behavior.

This study demonstrates the significant role of thromboxanes in mediating hemocyte aggregation upon infection foci of the fungal conidia in insects. Our working model of thromboxane actions at the cellular level is depicted in **Figure 9**. We expect to refine our model as new details emerge of continued research. Initially, the chemokine-like role of eicosanoids was introduced in another lepidopteran insect, *Manduca sexta*, in which hemocyte migration to the infection foci was inhibited by DEX treatment (18). Our current study suggests that thromboxanes act as chemokine-like factors to mediate hemocyte migration to infection foci during wound healing or other cellular immune responses in insects.

## Data Availability Statement

The datasets presented in this study can be found in online repositories. The names of the repository/repositories and accession number(s) can be found in the article/**Supplementary Material**.

## Author Contributions

MCR and YK carried out the experiment. MCR, DS, and YK wrote the manuscript with support from KN and JK. YK conceived the original idea. YK supervised the project. All authors contributed to the article and approved the submitted version.

## Funding

This work was supported by the National Research Foundation (NRF, grant number 2017R1A2133009815) of the Ministry of Science, ICT and Future Planning, Republic of Korea.

## Conflict of Interest

The authors declare that the research was conducted in the absence of any commercial or financial relationships that could be construed as a potential conflict of interest.

## Publisher’s Note

All claims expressed in this article are solely those of the authors and do not necessarily represent those of their affiliated organizations, or those of the publisher, the editors and the reviewers. Any product that may be evaluated in this article, or claim that may be made by its manufacturer, is not guaranteed or endorsed by the publisher.

## References

[1] Lemaitre B Hoffmann J. The Host Defense of *Drosophila Melanogaster* . Annu Rev Immunol (2007) 25:697–743. doi: 10.1146/annurev.immunol.25.022106.141615 17201680

[2] Kwon H Bang K Cho S. Characterization of the Hemocytes in Larvae of *Protaetia Brevitarsis Seulensis*: Involvement of Granulocyte-Mediated Phagocytosis. PloS One (2014) 9:e103620. doi: 10.1371/journal.pone.0103620 25083702 PMC4118905

[3] Lee J Hwang S Cho S. Immune Tolerance to an Intestine-Adapted Bacterium, *Chryseobacterium* Sp., Injected Into the Hemocoel of *Protaetia Brevitarsis Seulensis* . Sci Rep (2016) 6:31722. doi: 10.1038/srep31722 27530146 PMC4987663

[4] Kim H Choi D Jung J. Kim Y Eicosanoid Mediation of Immune Responses at Early Bacterial Infection Stage and its Inhibition by *Photorhabdus Temperata* Subsp. *Temperata*, an Entomopathogenic Bacterium. Arch Insect Biochem Physiol (2018) 99:e21502. doi: 10.1002/arch.21502 30120792

[5] Shrestha S Kim Y. Various Eicosanoids Modulate the Cellular and Humoral Immune Responses of the Beet Armyworm, *Spodoptera Exigua* . Biosci Biotechnol Biochem (2009) 73:2077–84. doi: 10.1271/bbb.90272 19734670

[6] Ishii K Hamamoto H Kamimura M Nakamura Y Noda H Imamura K. Insect Cytokine Paralytic Peptide (PP) Induces Cellular and Humoral Immune Responses in the Silkworm *Bombyx Mori* . J Biol Chem (2010) 285:28635–42. doi: 10.1074/jbc.M110.138446 PMC2937889 20622022

[7] Kim Y Ahmed S Stanley D An C. Eicosanoid-Mediated Immunity in Insects. Dev Comp Immunol (2018) 83:130–43. doi: 10.1016/j.dci.2017.12.005 29225005

[8] Stanley D Kim Y. Eicosanoid Signaling in Insects: From Discovery to Plant Protection. Crit Rev Plant Sci (2014) 33:20–63. doi: 10.1080/07352689.2014.847631

[9] Hasan MA Ahmed S Kim Y. Biosynthetic Pathway of Arachidonic Acid in *Spodoptera Exigua* in Response to Bacterial Challenge. Insect Biochem Mol Biol (2019) 111:103179. doi: 10.1016/j.ibmb.2019.103179 31255640

[10] Kim Y Stanley D. Eicosanoid Signaling in Insect Immunology: New Genes and Unresolved Issues. Genes (2021) 12:211. doi: 10.3390/genes12020211 33535438 PMC7912528

[11] Feletou M Huang Y Vanhoutte PM. Endothelium-Mediated Control of Vascular Tone: COX-1 and COX-2 Products. Br J Pharmacol (2011) 164:894–912. doi: 10.1111/j.1476-5381.2011.01276.x 21323907 PMC3195913

[12] Narumiya S Sugimoto Y Ushikubi F. Prostanoid Receptors: Structures, Properties, and Functions. Physiol Rev (1999) 79:1193–226. doi: 10.1152/physrev.1999.79.4.1193 10508233

[13] Braune S Küpper JH Jung F. Effect of Prostanoids on Human Platelet Function: An Overview. Int J Mol Sci (2020) 21:9020. doi: 10.3390/ijms21239020 PMC7730041 33260972

[14] Theopold U Krautz R Dushay MS. The *Drosophila* Clotting System and Its Messages for Mammals. Dev Comp Immunol (2014) 42:42–6. doi: 10.1016/j.dci.2013.03.014 23545286

[15] Hyrsl P Dobes P Wang Z Hauling T Wilhelmsson C Theopold U. Clotting Factors and Eicosanoids Protect Against Nematode Infections. J Innate Immun (2011) 3:65–70. doi: 10.1159/000320634 20948189

[16] Al Baki MA Roy CM Lee DH Stanley D Kim Y. The Prostanoids, Thromboxanes, Mediate Hemocytic Immunity to Bacterial Infection in the Lepidopteran *Spodoptera Exigua* . Dev Comp Immunol (2021) 120:104069. doi: 10.1016/j.dci.2021.104069 33737116

[17] Park J Kim Y. Phospholipase A_2_ Inhibitors in Bacterial Culture Broth Enhance Pathogenicity of a Fungus *Nomuraea Rileyi* . J Microbiol (2012) 50:644–51. doi: 10.1007/s12275-012-2108-3 22923114

[18] Merchant D Ertl RL Rennard SI Stanley DW Miller JS. Eicosanoids Mediate Insect Hemocyte Migration. J Insect Physiol (2008) 54:215–21. doi: 10.1016/j.jinsphys.2007.09.004 17996890

[19] Barletta ABF Trisnadi N Ramirez JL Barillas-Mury C. Mosquito Midgut Prostaglandin Release Establishes Systemic Immune Priming. iScience (2019) 19:54–62. doi: 10.1016/j.isci.2019.07.012 31351392 PMC6661395

[20] Shrestha S Kim Y. An Entomopathogenic Bacterium, *Xenorhabdus Nematophila*, Inhibits Hemocyte Phagocytosis of *Spodoptera Exigua* by Inhibiting Phospholipase A_2_ . J Invertebr Pathol (2007) 9:64–70. doi: 10.1016/j.jip.2007.02.009 17395196

[21] Ahmed S Stanley D Kim Y. An Insect Prostaglandin E_2_ Synthase Acts in Immunity and Reproduction. Front Physiol (2018) 9:1231. doi: 10.3389/fphys.2018.01231 30233407 PMC6131586

[22] Ahmed S Al Baki MA Lee J Seo DY Lee D Kim Y. The First Report of Prostacyclin and Its Physiological Roles in Insects. Gen Comp Endocrinol (2021) 301:113659. doi: 10.1016/j.ygcen.2020.113659 33166533

[23] Shrestha S Kim Y. Eicosanoids Mediate Prophenoloxidase Release From Oenocytoids in the Beet Armyworm, *Spodoptera Exigua* . Insect Biochem Mol Biol (2008) 38:99–112. doi: 10.1016/j.ibmb.2007.09.013 18070669

[24] Yajima M Takada M Takahashi N Kikuchi H Natori S Oshima Y. A Newly Established *In Vitro* Culture Using Transgenic *Drosophila* Reveals Functional Coupling Between the Phospholipase A_2_-Generated Fatty Acid Cascade and Lipopolysaccharide-Dependent Activation of the Immune Deficiency (Imd) Pathway in Insect Immunity. Biochem J (2003) 371:205–10. doi: 10.1042/bj20021603 PMC1223264 12513692

[25] Tootle TL Spradling AC. *Drosophila* Pxt: A Cyclooxygenase-Like Facilitator of Follicle Maturation. Development (2008) 135:839–47. doi: 10.1242/dev.017590 PMC2818214 18216169

[26] Kim Y Ahmed S Al Baki MA Kumar S Kim K Park Y. Deletion Mutant of PGE_2_ Receptor Using CRISPR-Cas9 Exhibits Larval Immunosuppression and Adult Infertility in a Lepidopteran Insect, *Spodoptera Exigua* . Dev Comp Immunol (2020) 111:103743. doi: 10.1016/j.dci.2020.103743 32464135

[27] Sajjadian SM Ahmed S Al Baki MA Kim Y. Prostaglandin D_2_ Synthase and its Functional Association With Immune and Reproductive Processes in a Lepidopteran Insect, *Spodoptera Exigua* . Gen Comp Endocrinol (2020) 287:113352. doi: 10.1016/j.ygcen.2019.113352 31794733

[28] Goh HG Lee SG Lee BP Choi KM Kim JH. Simple Mass-Rearing of Beet Armyworm, *Spodoptera Exigua* (Hübner) (Lepidoptera: Noctuidae), on an Artificial Diet. Korean J Appl Entomol (1990) 29:180–3.

[29] Humber RA. Identification of Entomopathogenic Fungi” in Manual of Techniques in Invertebrate Pathology. NY: Academic Press (2012). pp. 151–87.

[30] Vrain TC Wakarchuk DA Levesque AC Hamilton RI. Intraspecific rDNA Restriction Fragment Length Polymorphism in the *Xiphinema Americanum* Group. Fund Appl Nematol (1992) 15:563–74.

[31] Tamura K Stecher G Peterson D Filipski A Kumar S. MEGA6: Molecular Evolutionary Genetics Analysis Version 6.0. Mol Biol Evol (2013) 30:2725–9. doi: 10.1093/molbev/mst197 PMC3840312 24132122

[32] Winston PW Bates DH. Saturated Solutions for the Control of Humidity in Biological Research. Ecology (1960) 41:232–7. doi: 10.2307/1931961

[33] Roy MC Kim Y. Eicosanoid-Induced Calcium Signaling Mediates Cellular Immune Responses of *Tenebrio Molitor* . Entomol Exp Appl (2021) 169:888–98. doi: 10.1111/eea.13037

[34] Rohloff LH Wiesner A Giitz P. A Fluorescence Assay Demonstrating Stimulation of Phagocytosis by Haemolymph Molecules of *Galleria Mellonella* . J Insect Physiol (1994) 40:1045–9. doi: 10.1016/0022-1910(94)90057-4

[35] Vatanparast M Ahmed S Lee DH Hwang SH Hammock B Kim Y. EpOMEs Act as Immune Suppressors in a Lepidopteran Insect, *Spodoptera Exigua* . Sci Rep (2020) 10:20183. doi: 10.1038/s41598-020-77325-2 33214688 PMC7677322

[36] Bustin SA Benes V Garson JA Hellemans J Huggett J Kubista M. The MIQE Guidelines: Minimum Information for Publication of Quantitative Real-Time PCR Experiments. Clin Chem (2009) 55:611–22. doi: 10.1373/clinchem.2008.112797 19246619

[37] Livak KJ Schmittgen TD. Analysis of Relative Gene Expression Data Using Real Time Quantitative PCR and the 2^-ΔΔCT^ Method. Methods (2001) 25:402–8. doi: 10.1006/meth.2001.1262 11846609

[38] Vatanparast MD Ahmed S Herrero S Kim Y. A Non-Venomous Spla_2_ of a Lepidopteran Insect: Its Physiological Functions in Development and Immunity. Dev Comp Immunol (2018) 89:83–92. doi: 10.1016/j.dci.2018.08.008 30107251

[39] Bradford MM. A Rapid and Sensitive Method for the Quantitation of Microgram Quantities of Protein Utilizing the Principle of Protein-Dye Finding. Anal Biochem (1976) 72:248–54. doi: 10.1016/0003-2697(76)90527-3 942051

[40] SAS Institute Inc. SAS/STAT User’s Guide. Cary, NC: SAS Institute (1980).

[41] Litwin A Fedorowicz O Duszynska W. Characteristics of Microbial Factors of Healthcare-Associated Infections Including Multidrug-Resistant Pathogens and Antibiotic Consumption at the University Intensive Care Unit in Poland in the Years 2011-2018. Int J Environ Res Public Health (2020) 17:6943. doi: 10.3390/ijerph17196943 PMC7579392 32977435

[42] Mascarin GM Jaronski ST. The Production and Uses of *Beauveria Bassiana* as a Microbial Insecticide. World J Microbiol Biotechnol (2016) 32:1–26. doi: 10.1007/s11274-016-2131-3 27628337

[43] MacMillan D McCarron JG. The Phospholipase C Inhibitor U-73122 Inhibits Ca^2+^ Release From the Intracellular Sarcoplasmic Reticulum Ca^2+^ Store by Inhibiting Ca^2+^ Pumps in Smooth Muscle. Br J Pharm (2010) 160:1295–301. doi: 10.1111/j.1476-5381.2010.00771.x PMC2938802 20590621

[44] Chokshi R Fruasaha P Kozak JA. 2-Aminoethyl Dipheny Borinate (2-APB) Inhibits TRPM7 Channels Through in Intracellular Acidification Mechanism. Channels (2012) 6:362–9. doi: 10.4161/chan.21628 PMC3508775 22922232

[45] Kaja S Payne AJ Patel KR Naumchuk Y Koulen P. Differential Subcellular Ca^2+^ Signaling in a Highly Specialized Subpopulation of Astrocytes. Exp Neurol (2015) 265:59–68. doi: 10.1016/j.expneurol.2014.12.014 25542978 PMC4346429

[46] Lanner JT Georgiou DK Joshi AD Hamilton SL. Ryanodine Receptors: Structure, Expression, Molecular Details, and Function in Calcium Release. Cold Spring Harb Perspect Biol (2010) 2:a003996. doi: 10.1101/cshperspect.a003996 20961976 PMC2964179

[47] Willott E Hallberg CA Tran HQ. Influence of Calcium on *Manduca Sexta* Plasmatocyte Spreading and Network Formation. Arch Insect Biochem Physiol (2002) 49:187–202. doi: 10.1002/arch.10019 11921077

[48] Mortimer NT Goecks J Kacsoh BZ Mobley JA Bowersock GJ Taylor J. Parasitoid Wasp Venom SERCA Regulates *Drosophila* Calcium Levels and Inhibits Cellular Immunity. Proc Natl Acad Sci USA (2013) 110:9427–32. doi: 10.1073/pnas.1222351110 PMC3677475 23690612

[49] Ahmed S Seo K Kim Y. An Ovary-Specific Mucin is Associated With Choriogenesis Mediated by Prostaglandin Signaling in *Spodoptera Exigua* . Arch Insect Biochem Physiol (2021) 106:e21748. doi: 10.1002/arch.21748 33038048

[50] Gelosa P Ballerio R Banfi C Nobili E Gianella A Pignieri A. Terutroban, a Thromboxane/Prostaglandin Endoperoxide Receptor Antagonist, Increases Survival in Stroke-Prone Rats by Preventing Systemic Inflammation and Endothelial Dysfunction: Comparison With Aspirin and Rosuvastatin. J Pharmacol Exp Ther (2010) 334:199–205. doi: 10.1124/jpet.110.165787 20332187

[51] Habib A Fitzgerald GA Maclouf J. Phosphorylation of the Thromboxane Receptor Alpha, the Predominant Isoform Expressed in Human Platelets. J Biol Chem (1999) 274:2645–51. doi: 10.1074/jbc.274.5.2645 9915793

[52] Wikstrom K Kavanagh DJ Reid HM Kinsella BT. Differential Regulation of RhoA-Mediated Signaling by the TP Alpha and TP Beta Isoforms of the Human Thromboxane A_2_ Receptor: Independent Modulation of TP Alpha Signaling by Prostacyclin and Nitric Oxide. Cell Signal (2008) 20:1497–512. doi: 10.1016/j.cellsig.2008.04.006 PMC2681257 18502100

[53] Milatovic D Montine TJ Aschner M. Prostanoid Signaling: Dual Role for Prostaglandin E_2_ in Neurotoxicity. Neurotoxicology (2011) 32:312–9. doi: 10.1016/j.neuro.2011.02.004 PMC3090136 21376752

[54] Shrestha S Stanley D Kim Y. PGE_2_ Induces Oenocytoid Cell Lysis *via* a G Protein-Coupled Receptor in the Beet Armyworm, *Spodoptera Exigua* . J Insect Physiol (2011) 57:1568–76. doi: 10.1016/j.jinsphys.2011.08.010 21867708

[55] Jiang H Wei Z Nachman RJ Adams ME Park Y. Functional Phylogenetics Reveals Contributions of Pleiotropic Peptide Action to Ligand-Receptor Coevolution. Sci Rep (2014) 4:6800. doi: 10.1038/srep06800 25348027 PMC4210869

