# Supplementary material for: Thromboxane Mobilizes Insect Blood Cells to Infection Foci
Source: Front Immunol. 2021 Dec 20;12:791319. doi: 10.3389/fimmu.2021.791319 (PMC8720849; doi:10.3389/fimmu.2021.791319)
Supplement: Supplementary file 1 [file DataSheet_1.pdf]

SUPPLEMENTARY MATERIALS

Table S1. Primers used in this study

Table S2. Identification of an EPF isolate using ITS sequence. The sequence region (754 nucleotide (nt)) contains partial 18S rRNA (153 nt), ITS1 (183 nt), 5.8S rRNA (156 nt), ITS2 (156 nt), and partial 28S rRNA (106 nt).

Table S3. Insecticidal activities of *M. rileyi* against three different insect species. Fourth instar larvae of *S. exigua* and third instar larvae of *P. xylostella* were used in bioassays. Larvae of a coleopteran species, *T. molitor*, with body length of approximately 3.5 cm were used in bioassays. It was replicated three times with 10 insects per replications. Median lethal times (LT_{50}s) were estimated as fungal concentration of 1,000 conidia per larvae.

Table S4. GenBank accession numbers of putative G protein-coupled receptors (GPCRs) of *S. exigua*

Figure S1. Identification and pathogenicity of the fungal isolate. (A) Morphological identification of *M. rileyi* isolate based on colony, conidiophore, and conidial shapes. (B) Molecular identification of *M. rileyi* isolate. Sequence analysis of ITS/rRNA region of the fungal isolate. Two ITS regions are marked with boxes. The DNA sequence was deposited to GenBank with an accession number of MT835256.1. (C) Phylogenetic relationship of the fungal isolate with other fungal species based on ITS/rRNA sequences. The tree was constructed with the Neighbor-joining method using MEGA6.0. Bootstrapping values on branches were obtained with 1,000 repetitions. Genbank accession numbers are FJ824809.1

for *Metharizium rileyi*, AB214654.1 for *Metacordyceps chlamydosporia*, AB100362.1 for *Cordyceps chlamydosporia*, AB214658.1 for *Pochonia suchlasporia*, MH864644.1 for *Verticillium cephalosporum*, AB102783.1 for *Neotyphodium uncinatum*, HM534896.1 for *Nectria eustromatica*, AB208109.1 for *Blistum tomentosum*, KC436057.1 for *Claviceps purpurea*, AJ301993.1 for *Myrothecium roridum*, and AB258369.1 for *Paecilomyces carneus*. (D) Pathogenic activity of the fungal isolate was determined by leaf (2 cm^2) dipping method applying 1,000 conidia per larva to filter paper in Petri dish containing individual larvae. The experimental unit (=Petri dish) was replicated 30 times and experiment was replicated thrice. Mortality data was recorded every day up to 7 days. Dead insects were checked under microscope.

Figure S2. Differential virulence activities of isolated fungi on insect host cuticle defense system. (A) Insecticidal activity of fungal isolate on newly molted larvae of *S. exigua*. Newly molted fourth instar larvae were considered as unsclerotized and one day post molted larvae were treated as sclerotized. 1,000 conidia per larva were used for topical application. (B) Virulence activity of isolated fungi based on cuticle barrier. To remove cuticle barrier (DeEpi), 100% acetone was used. Experiment was replicated three times containing 10 larvae per replication. 1,000 conidia per larva were used. Mortality data was taken up to 5 days. Asterisk above standard error bars indicate significant difference among means at Type I error = 0.05 (LSD test).

Figure S3. Induction of calcium signal in response to different prostaglandins. Fifth instar larvae of *S. exigua* were injected with 2 µL of Fura-8 (1 mM). After 30 min incubation, 1µg of PGE_2 (**Figure S3A**), PGD_2 (**Figure S3B**), PGI_2 (**Figure S3C**), TXA_2 (**Figure S3D**) and TXB_2 (**Figure S3E**) was injected. At different time points, hemolymph was collected and

fixed with 2.5% paraformaldehyde. Time-course of fura-positive cells was counted. Micro-aggregation of hemocytes was counted from randomly chosen 100 cells. Each time point was independently replicated three times. Different letters above standard error bars indicate significant difference at Type I error = 0.05 (LSD test).

Figure S4. LC-MS/MS chromatogram of fat bodies sample containing TXB_2 confirmed by two specific MS/MS fragments (169 Da and 195 Da) in 369 Da primary fragments.

Table S1. Primers used in this study

Genes	Primer sequences (5' - 3')	Temp[1] (°C)	Purposes
SeTXAS	CAAGATGCGCCTAATGGTGC	50	RT-PCR RT-qPCR
	ATGCCGACGATGATGACTCC		
	TAATACGACTCACTATAGGGAGACAAGATGCGCCTAATGGTGC	50	RNAi
	TAATACGACTCACTATAGGGAGAATGCCGACGATGATGACTCC		
Se-sPLA$_2$	GCGGAAGCGAGTACCTTATT	50	RT-qPCR
	CTTTCTCTTCGCCTAGGTCATC		
Se-iPLA$_2$A	ATGTACAGTTTATGTCGGGCT	50	RT-qPCR
	GAGCACGTCGCTCAGTATC		
Se-iPLA$_2$B	TCGAGCATCTGCACCACTAC	50	RT-qPCR
	GACGACGCCATTTATCTCCTTAG		
SePOX-A	CCAAGCTTGTAGCGCCCATAG	52	RT-qPCR
	GCCCAATGTACCTCCTTGC		
SePOX-F	CGACAACATCGCAACTGTTC	50	RT-qPCR
	TGGTTCACACGAATGTCACC		
SePOX-H	CGTCTAGAACTTTCTGCGTG	50	RT-qPCR
	GACCAGGCAAACTTCTCATG		
Se-PGE$_2$R	AAGGCCCTTCCTCTACCAAA	52	RT-PCR RT-qPCR
	AGATCATGAAGAGCACGGAGAG		
	TAATACGACTCACTATAGGGAGAAAGGCCCTTCCTCTACCAAA	52	RNAi
	TAATACGACTCACTATAGGGAGAAGATCATGAAGAGCACGGAGAG		
RL32	ATGCCCAACATTGGTTACGG	52	RT-PCR RT-qPCR
	TTCGTTCTCCTGGCTGCGGA		

[1] ‘Temp’ indicates annealing temperature.

Table S2. Identification of an entomopathogenic fungal isolate using ITS sequence (GenBank accession number: MT835256.1). The sequence region (754 nucleotide (nt)) contains partial 18S rRNA (153 nt), ITS1 (183 nt), 5.8S rRNA (156 nt), ITS2 (156 nt), and partial 28S rRNA (106 nt).

Blast match	Total score	E value	GenBank accession number	Identity (%)
Metarhizium rileyi isolate	1,375	0.0	FJ824809.1	99.60
Metarhizium rileyi strain MAFF 830007	1,373	0.0	AB268359.1	99.73
Metarhizium rileyi	1,363	0.0	AB100361.1	99.60
Metarhizium rileyi strain DT2011N7	1,123	0.0	KX641194.1	99.36
Metarhizium rileyi strain Nr101	1,118	0.0	KY436756.1	99.19

Table S3. Insecticidal activities of *M. rileyi* against three different insect species. Fourth instar larvae of *S. exigua* and third instar larvae of *P. xylostella* were used in bioassays. Larvae of a coleopteran species, *T. molitor*, with body length of approximately 3.5 cm were used in bioassays. It was replicated three times with 10 insects per replication. Median lethal times (LT_{50}s) were estimated from a treatment using a fungal concentration of 1,000 conidia per larva.

**Target insects**	**N**	**LT_{50} (95% CI), h**	**Slope ± SE**	**χ^2**	**df**
T. molitor	30	182.65 (147.3 - 226.5) a[1]	4.34 ± 0.05	0.942	3
P. xylostella	30	136.35 (110.1 - 168.8) ab	3.81 ± 0.05	0.978	4
S. exigua	30	111.41 (93.3 -132.9) b	4.64 ± 0.04	0.983	4

[1] Different letters following LT50 values represent significant difference by non-overlapping of 95% CI.

Table S4. GenBank accession numbers of putative G protein-coupled receptors (GPCRs) of *S. exigua*

GPCR	Annotated genes	GenBank accession numbers of *S. exigua*		GenBank accession numbers of References	
		TSA	NR	Species	NR
GPCR1	Leucine-rich repeat	GGRZ01069035.1	MZ501834	*Bombyx mori*	NP_001037033.1
GPCR2	5-hydroxytryptamine /serotonin receptor	-	AZA07970.1	*Spodoptera exigua*	-
GPCR3	Dopamine receptor 2-like	GGRZ01048721.1	MZ501835	*Spodoptera litura*	XP_022834024.1
GPCR4	Dopamine receptor 1-like	GGRZ01057920.1	MZ501858	*Spodoptera litura*	XP_022827490.1
GPCR5	Dopamine N-acetyltransferase-like	GGRZ01138956.1	MZ501836	*Spodoptera litura*	XP_022826743.1
GPCR6	Octopamine receptor beta-1R-like	GGRZ01102221.1	MZ501837	*Spodoptera litura*	XP_022827519.1
GPCR7	Octopamine receptor beta-3R-like	GGRZ01084708.1	MZ501838	*Spodoptera litura*	XP_022827237.1
GPCR8	Octopamine receptor 1-like	GGRZ01092489.1	MZ501839	*Spodoptera litura*	XP_022827626.1
GPCR9	Octopamine receptor beta-2R	GGRZ01087211.1	MZ501840	*Spodoptera litura*	XP_022827354.1
GPCR10	Allatotropin	-	AEO27700.1	*Spodoptera exigua*	-
GPCR11	Sex peptide receptor-like	GARL01043383.1	MZ501841	*Spodoptera litura*	XP_022816105.1
GPCR12	Myosuppressin	-	AXY04275.1	*Spodoptera exigua*	-
GPCR13	Corazonin	-	AXY04245.1	*Spodoptera exigua*	-
GPCR14	Tachykinin	-	AXY04299.1	*Spodoptera exigua*	-
GPCR15	Capability/ Cardioacceleratory	-	AXY04240.1	*Spodoptera exigua*	-
GPCR16	FMRFamide	-	AXY04254.1	*Spodoptera exigua*	-
GPCR17	SIFamide	-	AXY04297.1	*Spodoptera exigua*	-
GPCR18	Diuretic hormone 45	-	AXY04251.1	*Spodoptera exigua*	-
GPCR19	Corticotropin-releasing factor-like-diuretic hormone 44	-	AXY04246.1	*Spodoptera exigua*	-
GPCR20	Calcitonin gene-related peptide type 1 receptor-like	GGRZ01038487.1	MZ501842	*Spodoptera litura*	XP_022838045.1
GPCR21	Latrophilin Cirl isoform X5	GGRZ01037013.1	MZ501843	*Spodoptera litura*	XP_022834058.1
GPCR22	G-protein coupled receptor Mth2-like	GARL01065745.1	MZ501844	*Spodoptera litura*	XP_022813850.1
GPCR23	G-protein coupled receptor Mth2-like isoform X7	GARL01040814.1	MZ501845	*Spodoptera litura*	XP_022834098.1
GPCR24	G-protein coupled receptor Mth2-like isoform X1	GGRZ01082913.1	MZ501846	*Spodoptera litura*	XP_022814039.1
GPCR25	Probable G-protein coupled receptor Mth-like 3	GGRZ01068498.1	MZ501847	*Spodoptera litura*	XP_022817499.1
GPCR26	Probable G-protein coupled receptor Mth-like 5	GGRZ01117535.1	MZ501848	*Spodoptera litura*	XP_022817498.1
GPCR27	GABA-B receptor type 1	GGRZ01069701.1	MZ501849	*Heliothis virescens*	CDF56917.1
GPCR28	Frizzled-7-B	GGRZ01224538.1	MZ501850	*Spodoptera litura*	XP_022821197.1
GPCR29	Frizzled-4	GGRZ01247645.1	MZ501851	*Spodoptera litura*	XP_022823006.1
GPCR30	Frizzled-2 isoform X3	GGRZ01215736.1	MZ501852	*Trichoplusia ni*	XP_026733990.1

GPCR31	Frizzled-10-like	GGRZ01095571.1	MZ501853	*Spodoptera litura*	XP_022822728.1
GPCR32	Protein smoothened-like isoform X2	GGRZ01137765.1	MZ501854	*Spodoptera litura*	XP_022822897.1
GPCR33	Moody	GGRZ01106723.1	MZ501855	*Spodoptera litura*	XP_022826488.1
GPCR34	No18	GGRZ01113408.1	MZ501856	*Spodoptera litura*	XP_022826898.1
GPCR35	158	GGRZ01069261.1	MZ501857	*Spodoptera litura*	XP_022820928.1
Se-hcPGGPCR1	hcPGGPCR1	-	ADE43129.1	*Spodoptera exigua*	-
Se-PGE$_2$R	Prostaglandin E$_2$ receptor	-	QEN91980.1	*Spodoptera exigua*	-
Hs-TXA$_2$R	Thromboxane A$_2$ receptor isoform alpha	-	-	*Homo sapiens*	NP_001051.1
Mm-TXA$_2$R	Thromboxane A$_2$ receptor	-	-	*Mus musculus*	NP_001345441.1
Dr-TXA$_2$R	Thromboxane A$_2$ receptor	-	-	*Danio rerio*	NP_001272464.1

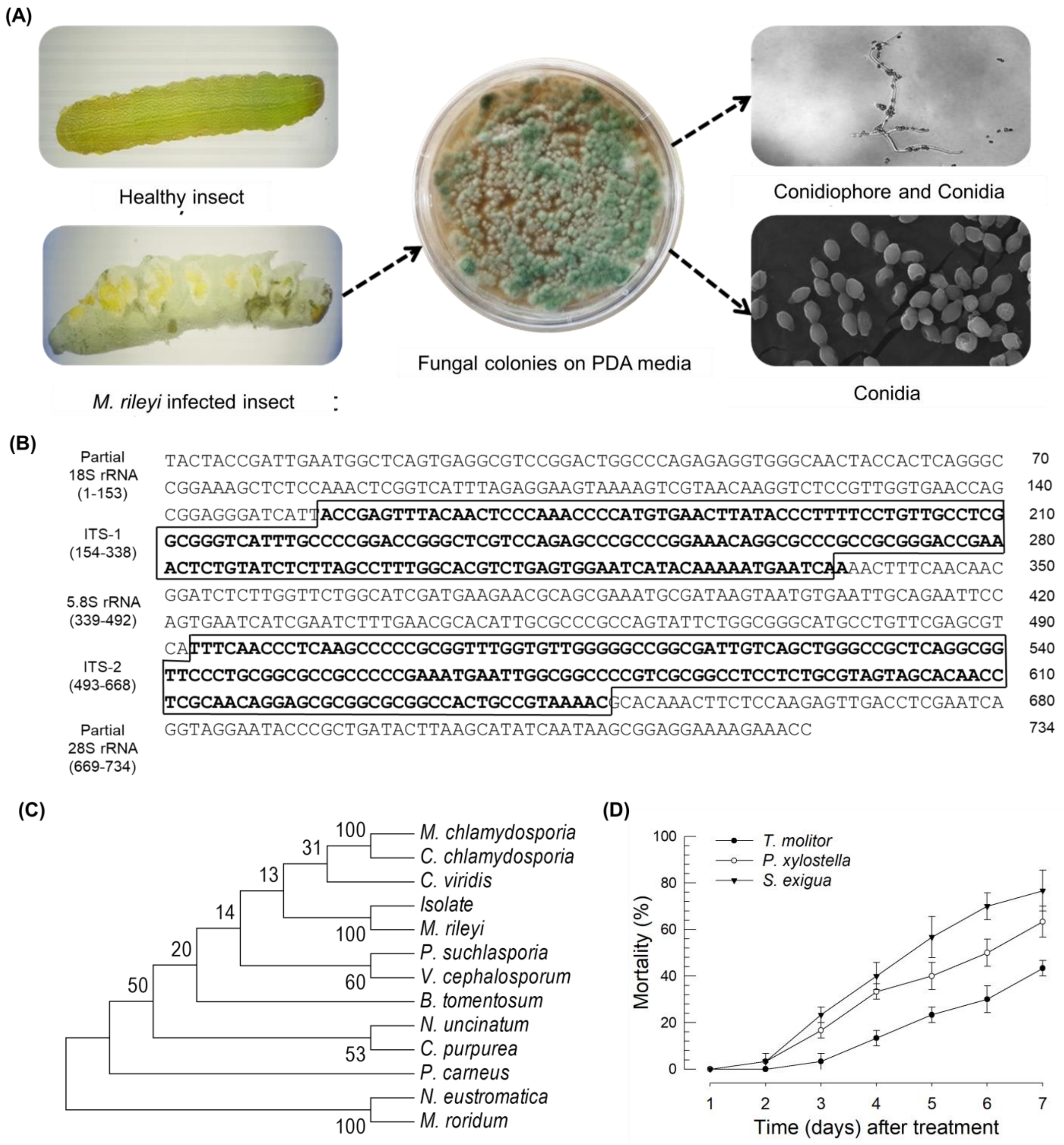
(A)
Healthy insect
M. rileyi infected insect
Fungal colonies on PDA media
Conidiophore and Conidia
Conidia
(B)
Partial 18S rRNA (1-153)
ITS-1 (154-338)
5.8S rRNA (339-492)
ITS-2 (493-668)
Partial 28S rRNA (669-734)
TACTACCGATTGAATGGCTCAGTGAGGCGTCCGGACTGGCCCAGAGAGGTGGGCAACTACCACTCAGGGC 70
CGGAAAGCTCTCCAAACTCGGTCATTTAGAGGAAGTAAAAGTCGTAACAAGGTCTCCGTTGGTGAACCAG 140
CGGAGGGATCATTACCGAGTTTACAACTCCCAAACCCCATGTGAACTTATACCCTTTTCCTGTTGCCTCG 210
GCGGGTCATTTGCCCCGGACCGGGCTCGTCCAGAGCCCGCCCGGAAACAGGCGCCCGCCGCGGACCGAA 280
ACTCTGTATCTCTTAGCCTTTGGCACGTCTGAGTGGAATCATACAAAAATGAATCAAAACTTTCAACAAC 350
GGATCTCTTGGTTCTGGCATCGATGAAGAACGCAGCGAAATGCGATAAGTAATGTGAATTGCAGAATTCC 420
AGTGAATCATCGAATCTTTGAACGCACATTGCGCCCGCCAGTATTCTGGCGGGCATGCCTGTTCGAGCGT 490
CATTTCAACCCTCAAGCCCCCGCGGTTTGGTGTTGGGGGCCGGCGATTGTCAGCTGGGCCGCTCAGGCGG 540
TTCCCTGCGGCGCCGCCCCCGAAATGAATTGGCGGCCCCGTCGCGGCCTCCTCTGCGTAGTAGCACAACC 610
TCGCAACAGGAGCGCGGCGCGGCCACTGCCGTAAAACGCACAAACTTCTCCAAGAGTTGACCTCGAATCA 680
GGTAGGAATACCCGCTGATACTTAAGCATATCAATAAGCGGAGGAAAAGAAACC 734
(C)
M. chlamydosporia
C. chlamydosporia
C. viridis
Isolate
M. rileyi
P. suchlasporia
V. cephalosporum
B. tomentosum
N. uncinatum
C. purpurea
P. carneus
N. eustromatica
M. roridum
100
31
13
14
100
20
60
50
53
100
(D)
T. molitor
P. xylostella
S. exigua
Mortality (%)
0
20
40
60
80
100
1
2
3
4
5
6
7
Time (days) after treatment

Figure S1

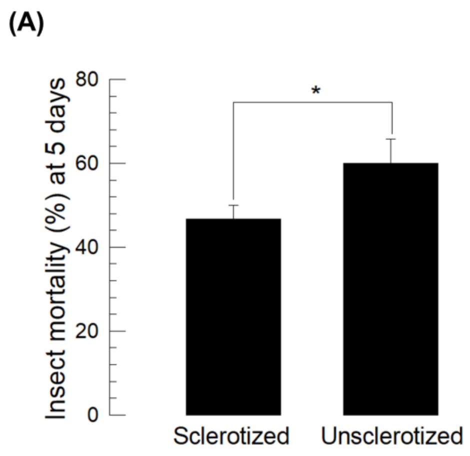
(A)
Insect mortality (%) at 5 days
80
60
40
20
0
*
Sclerotized
Unsclerotized
(B)
Insect mortality (%) at 5 days
100
80
60
40
20
0
*
Epi
DeEpi

Figure S2

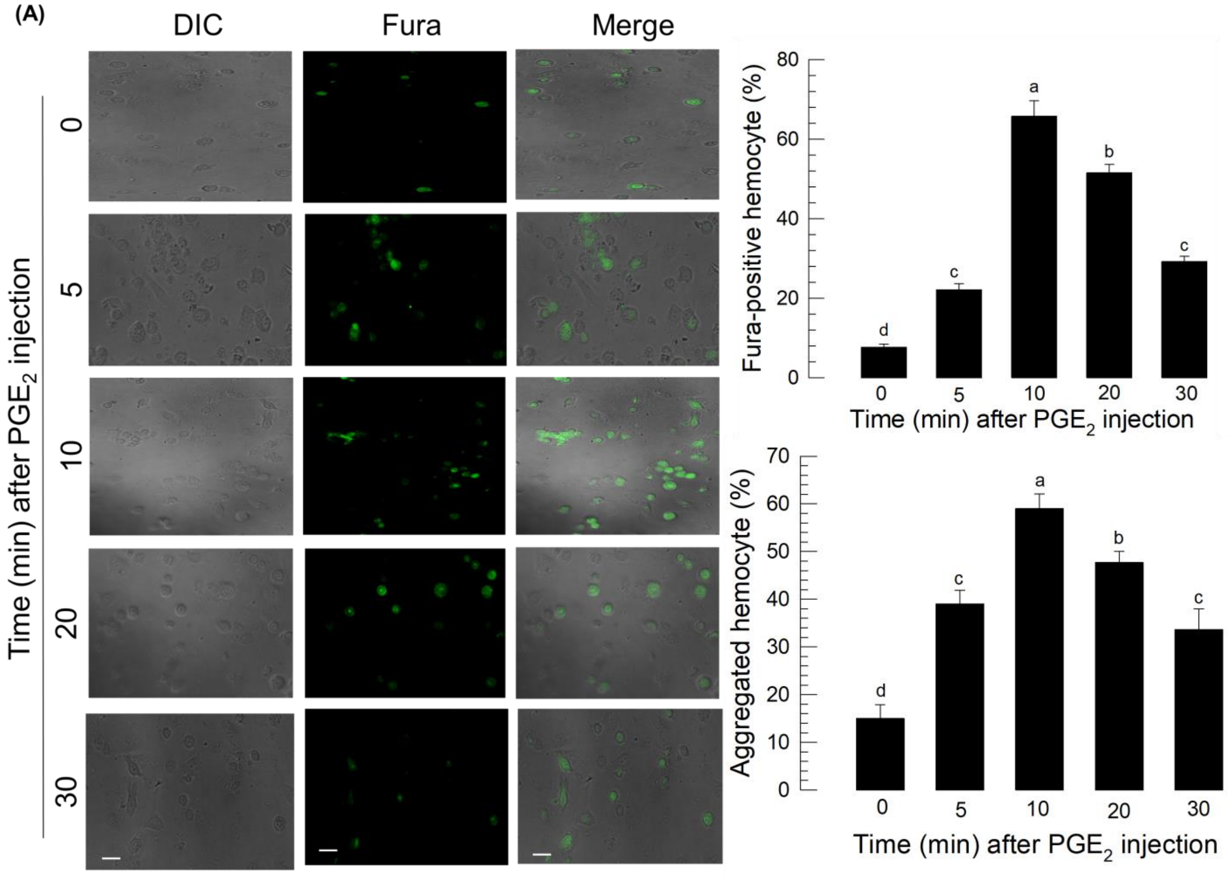
(A)
DIC
Fura
Merge
Time (min) after PGE2 injection
0
5
10
20
30
Fura-positive hemocyte (%)
Aggregated hemocyte (%)
a
b
c
d
Time (min) after PGE2 injection

Figure S3A

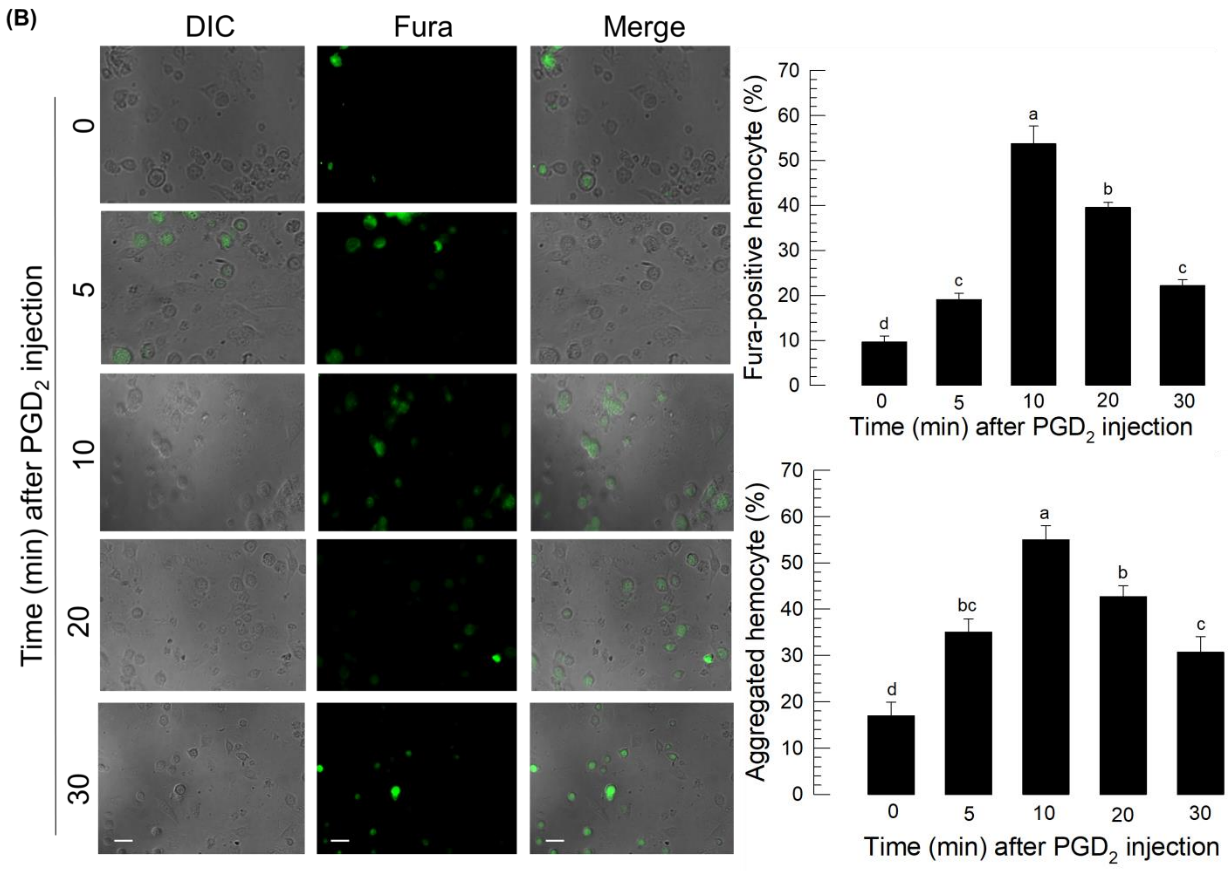
(B)
DIC
Fura
Merge
Time (min) after PGD2 injection
0
5
10
20
30
Fura-positive hemocyte (%)
Time (min) after PGD2 injection
Aggregated hemocyte (%)
Time (min) after PGD2 injection
a
b
c
d
bc

Figure S3B

(C)

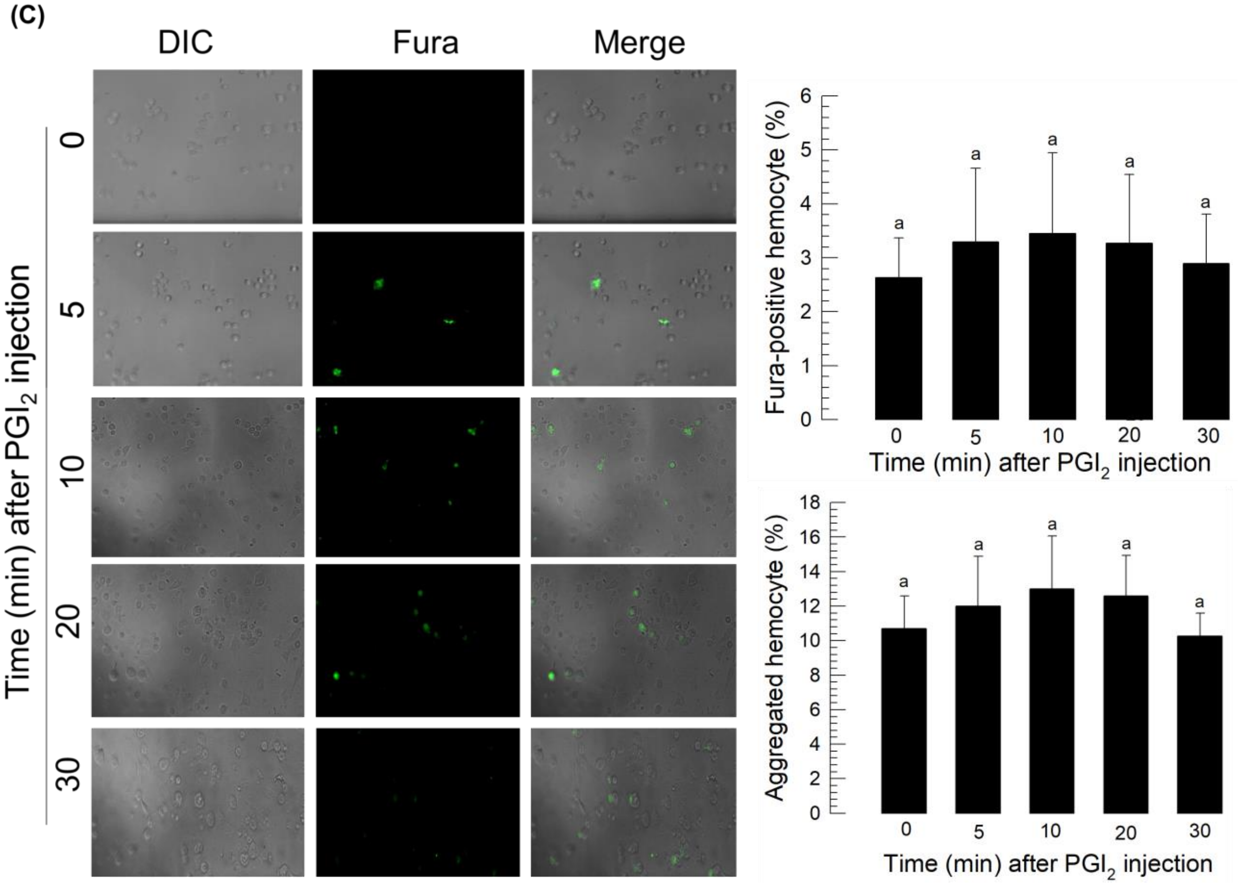
DIC
Fura
Merge
Time (min) after PGI_2 injection
0
5
10
20
30
Fura-positive hemocyte (%)
0
1
2
3
4
5
6
a
a
a
a
a
0
5
10
20
30
Time (min) after PGI_2 injection
Aggregated hemocyte (%)
0
2
4
6
8
10
12
14
16
18
a
a
a
a
a
0
5
10
20
30
Time (min) after PGI_2 injection

Figure S3C

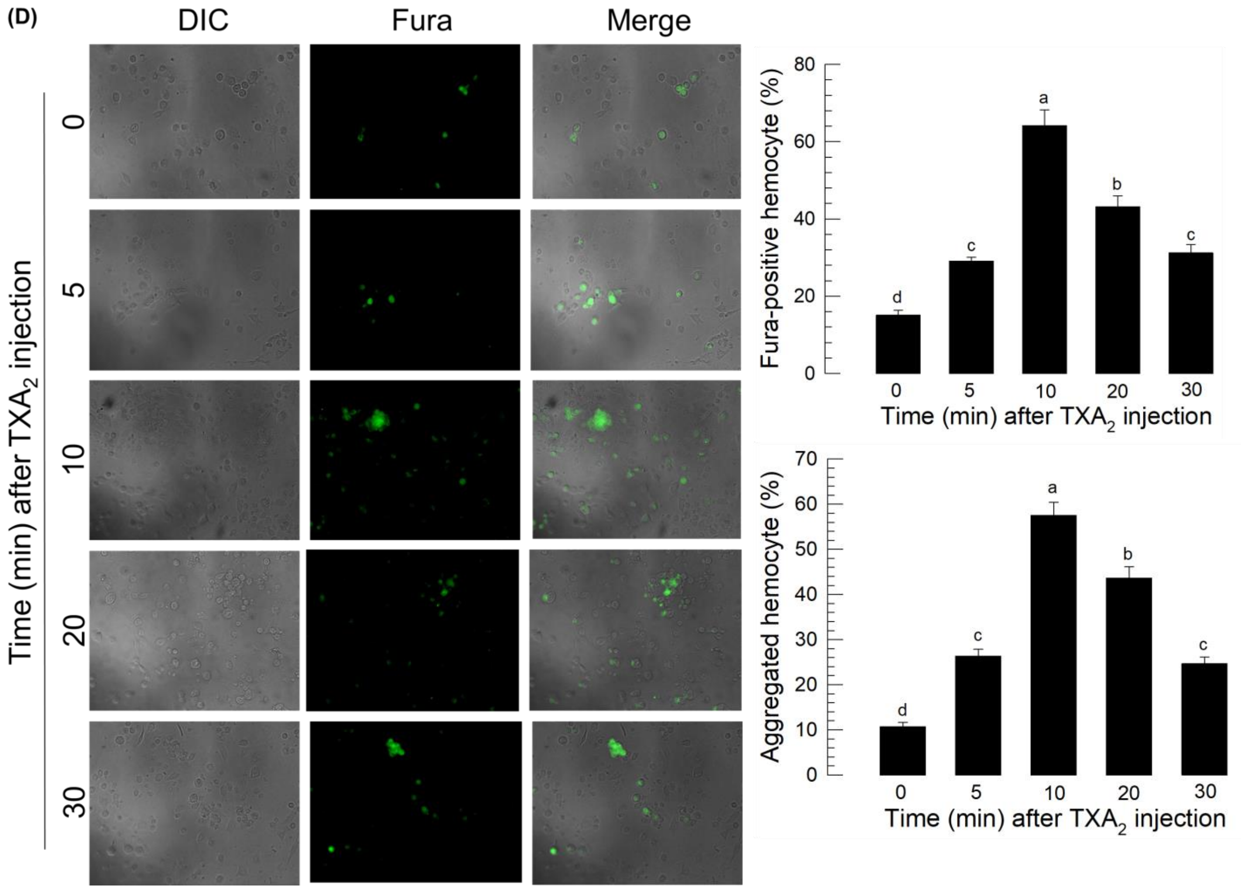

(D)
DIC
Fura
Merge
Time (min) after TXA2 injection
0
5
10
20
30
Fura-positive hemocyte (%)
Time (min) after TXA2 injection
Aggregated hemocyte (%)
Time (min) after TXA2 injection

Figure S3D

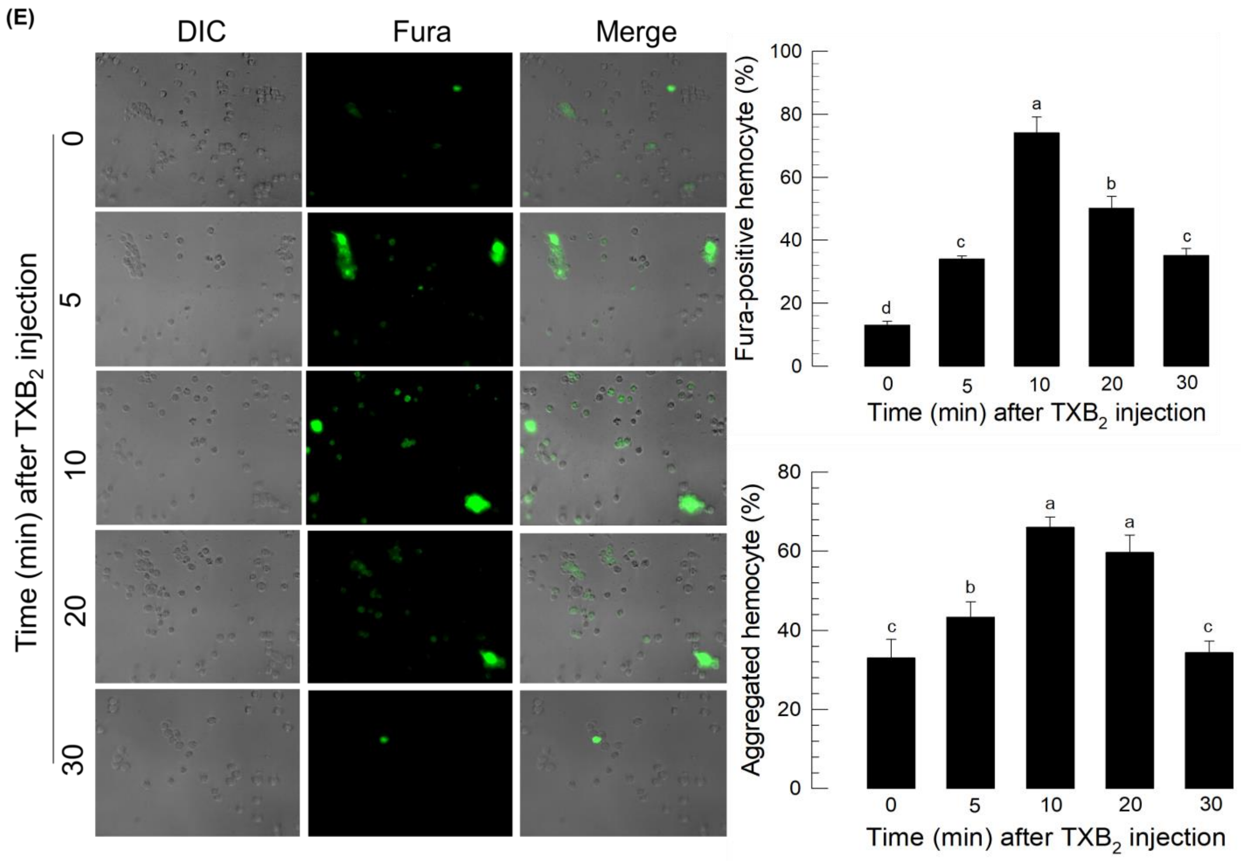
(E)
DIC
Fura
Merge
Time (min) after TXB2 injection
0
5
10
20
30
Fura-positive hemocyte (%)
100
80
60
40
20
0
d
c
a
b
c
0
5
10
20
30
Time (min) after TXB2 injection
Aggregated hemocyte (%)
80
60
40
20
0
c
b
a
a
c
0
5
10
20
30
Time (min) after TXB2 injection

Figure S3E

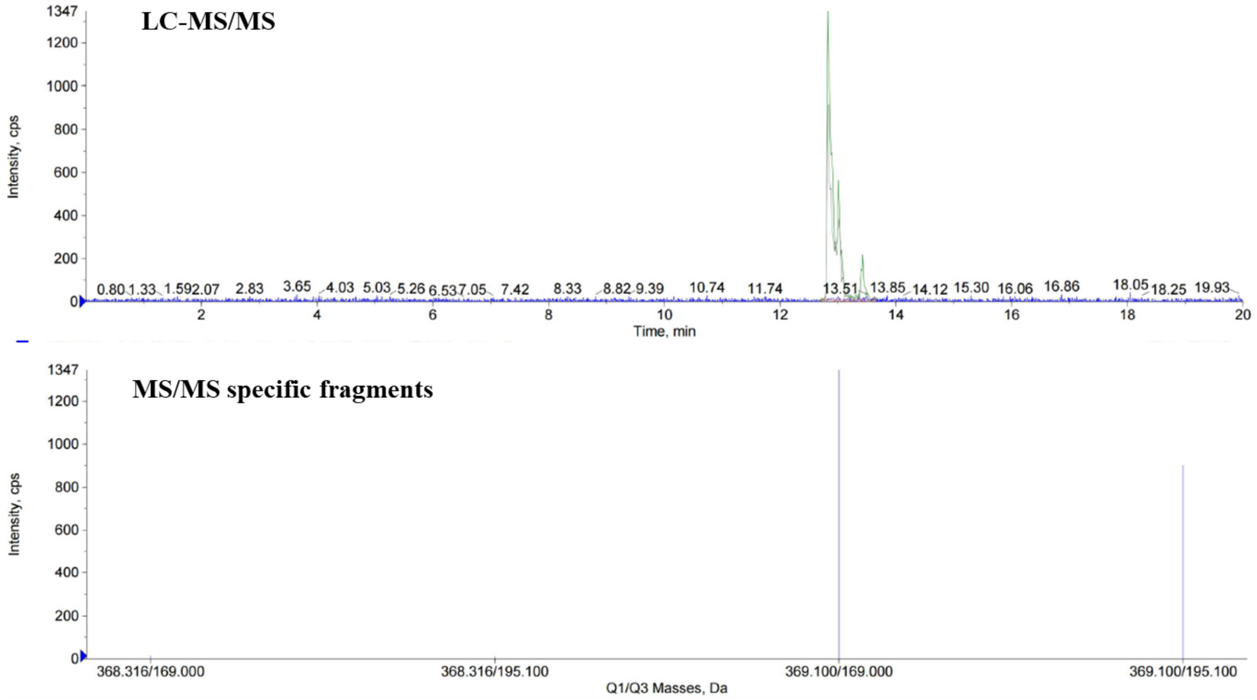
LC-MS/MS
Intensity, cps
Time, min
0.80 1.33 1.59 2.07 2.83 3.65 4.03 5.03 5.26 6.53 7.05 7.42 8.33 8.82 9.39 10.74 11.74 13.51 13.85 14.12 15.30 16.06 16.86 18.05 18.25 19.93
MS/MS specific fragments
Intensity, cps
Q1/Q3 Masses, Da
368.316/169.000
368.316/195.100
369.100/169.000
369.100/195.100

Figure S4
